# Failure Mode and Effect Analysis Using Large-Scale Group Decision Making and Normal Cloud Model

**DOI:** 10.3390/e28030360

**Published:** 2026-03-22

**Authors:** Lijie Wu, Changchun Liu, Hanwen Song

**Affiliations:** 1 Department of Sino-German Engineer, Shanghai Technical Institute of Electronics and Information, Shanghai 201400, China; 2 Department of Aerospace and Mechanics, Tongji University, Shanghai 200092, China

**Keywords:** failure modes and effects analysis, large-scale group decision making, normal cloud models, comprehensive weight, heterogeneous data

## Abstract

Failure Modes and Effects Analysis (FMEA) is crucial for complex system reliability. However, traditional FMEA and its existing enhancements face significant limitations. These notably include difficulties in handling diverse heterogeneous data, effectively coordinating large expert groups, and robustly propagating inherent uncertainties. To bridge these critical gaps, this paper proposes an innovative and robust FMEA framework, specifically designed for Large Group Decision Making (LGDM) under uncertainty, leveraging the Normal Cloud Model (NCM). First, LGDM is genuinely integrated into FMEA by involving an unprecedented number of experts (>50). Second, a broad spectrum of heterogeneous data, including exact numbers, interval numbers, NCMs, linguistic terms, and linguistic expressions, is utilized to effectively model and manage diverse uncertainties. Third, a four-step data preprocessing method is incorporated to efficiently screen invalid and low-quality inputs, significantly enhancing the reliability of aggregated results. Fourth, an innovative and comprehensive expert weight determination method that judiciously combines subjective factors with objective data quality is proposed, ensuring more trustworthy and equitable aggregation of judgments. Distinctively, our method explicitly preserves and propagates uncertainty information across the entire computational process, yielding more insightful and informative results beyond simple rankings, encompassing detailed quantitative uncertainty analysis. A practical case study, alongside detailed result analysis, sensitivity analysis, both qualitative and quantitative comparative analysis, and advantages and limitations analysis, collectively confirms the effectiveness, practicality, rationality, and robustness of the proposed method. The sensitivity analyses demonstrate that the final risk rankings are highly stable even under varying trade-off coefficients, confirming the method’s strong robustness and insensitivity to parameter fluctuations. Our framework provides a scientifically advanced and robust approach for FMEA in complex decision-making environments, particularly applicable to high-stakes industries such as modern aviation, thereby enabling more informed risk management decisions.

## 1. Introduction

Proactively identifying and prioritizing Failure Modes (FMs) is critical for the reliability and safety of complex industrial systems. Failure Modes and Effects Analysis (FMEA), a cornerstone risk evaluation methodology developed by NASA in the 1960s [1], remains widely adopted across high-risk sectors to systematically evaluate and prioritize FMs based on their potential impact.

FMEA employs a systematic multi-step process, utilizing the Risk Priority Number (RPN) to quantify and rank FMs. The RPN is calculated as O × S × D [2], where O is occurrence, S is severity, and D is probability of non-detection, typically scaled from 1 to 10. A higher RPN indicates a greater risk and demands higher prioritization.

However, the traditional FMEA approach is increasingly inadequate for modern industrial settings, which are often characterized by complex decision making involving large groups of experts where data is inherently heterogeneous and uncertain [3]. The profound and well-documented limitations of traditional FMEA in such environments are summarized as follows:

(1) Subjectivity and Intrinsic Uncertainty: Expert judgments are inherently subjective and frequently expressed imprecisely using linguistic terms (e.g., “high,” “moderate”). Representing these nuanced evaluations with exact numerical scores for all FMs and Risk Factors (RFs) oversimplifies the inherent uncertainty and semantic richness of expert opinions. Thus, the effective modeling and processing of diverse uncertain information are crucial.

(2) Scalability Challenges in LGDM: Modern complex systems necessitate input from numerous experts across various domains. However, most existing FMEA models assume homogeneous participation and small-scale input, failing to accommodate the flexibility required for large-scale, diverse, or selective expert engagement. The genuine integration of LGDM into FMEA remains scarce.

(3) Heterogeneous Data and Inconsistencies: Experts may provide invalid, incomplete, or low-quality evaluation data due to varying expertise levels or fatigue. Without robust preprocessing mechanisms, these unreliable inputs can severely distort the final risk rankings.

(4) Inadequate Weighting Mechanisms: Assigning equal weights to all experts ignores crucial differences in domain expertise and data quality, leading to biased or untrustworthy aggregation. A scientific weighting model accounting for varied expertise and data quality is needed.

(5) Limited Propagation of Uncertainty: While many advanced FMEA methods attempt to model uncertainty in individual inputs, they often fail to propagate this uncertainty coherently throughout the entire decision-making process. This results in “black-box” outcomes where the final risk prioritization lacks clear interpretability regarding its inherent uncertainty.

To address the limitations of subjectivity and uncertainty, recent advancements have introduced sophisticated mathematical tools such as fuzzy sets [4,5,6], interval type-2 trapezoidal fuzzy numbers [7], linguistic variables [8,9], and cloud models [10,11,12,13]. Of particular note, the Normal Cloud Model (NCM) effectively converts qualitative expert judgments into quantitative data, while preserving their inherent fuzziness and randomness. This capability has spurred the development of several hybrid decision-making approaches that build upon the NCM. Prominent examples include the Rough Fuzzy Integrated Clouds (RFICs) paired with Technique for Order of Preference by Similarity to Ideal Solution (TOPSIS) [14,15], the NCM combined with the VlseKriterijumska Optimizacija I Kompromisno Resenje (VIKOR) method [16], the interval rough cloud optimization model [17], and the NCM combined with Decision-Making Trial and Evaluation Laboratory (DEMATEL) method [18].

Despite these valuable contributions, a unified, robust, and practical FMEA framework that simultaneously addresses LGDM scalability, heterogeneous data processing, dynamic expert weighting, and end-to-end uncertainty propagation, specifically tailored for real-world industrial applications, remains an open research challenge. The limitations outlined above are deeply interconnected, each compounding the effects of the others. For example, the inherent subjectivity and uncertainty of expert judgments are significantly amplified in LGDM settings. If left unaddressed, issues of heterogeneous data quality or improperly weighted expert inputs can further distort the final aggregated results, potentially undermining their reliability. Consequently, effectively tackling these multi-faceted challenges requires more than incremental adjustments to isolated methodologies; it demands a holistic, integrated framework where multiple specialized techniques operate in synergy. An isolated or piecemeal approach—such as applying only an uncertainty model or only a weighting scheme—would inevitably leave critical gaps unaddressed. To this end, the present paper proposes a novel and comprehensive solution: the NCM-LGDM-FMEA framework. This framework systematically integrates four key components: (1) the NCM as a unified mathematical representation to capture and propagate uncertainty throughout the entire process; (2) a robust data preprocessing pipeline to cleanse and validate large-scale, heterogeneous inputs; (3) a dynamic dual-factor weighting algorithm that balances expert authority with data credibility; and (4) a scalable LGDM architecture to organize the overall workflow. This integrated design ensures that the strength of each component compensates for the operational contexts of the others, thereby creating a coherent and comprehensive solution where the whole is demonstrably greater than the sum of its parts.

The key contributions of this research are summarized as follows.

(1) A Scalable LGDM Architecture: Our proposed NCM-LGDM-FMEA framework supports a significantly larger number of experts, allowing for flexible and selective participation in evaluating only the subset of FMs and RFs relevant to their specific expertise. This dramatically enhances practicality and applicability in real-world industrial environments.

(2) A Four-Step Robust Data Preprocessing Pipeline: We introduce an automated, four-step data preprocessing method to identify and filter out invalid and low-quality evaluation data from large group experts. This crucial step ensures the integrity and reliability of all subsequent aggregated results.

(3) Uncertainty Modeling via NCM: Unlike static or local uncertainty modeling in prior methods, our NCM-based approach captures randomness and fuzziness across the entire computational chain—from input to output. This ensures more consistent, informative, and semantically meaningful output.

(4) A Novel Dynamic Expert Weighting Algorithm: We propose a novel dual-factor weighting algorithm that dynamically and comprehensively assesses each expert’s importance. It considers both their intrinsic identity (professional title and work experience) and the quality of their provided data (Uncertainty Degree (UD) and Difference Degree (DD)). This mechanism prevents biases introduced by overconfident or less informed contributors, leading to fairer and more accurate aggregations.

(5) Comprehensive Validation and Comparative Analysis: We conduct extensive experimental validation using a practical case study. This includes detailed result analysis, sensitivity analysis, rigorous qualitative and quantitative comparative analysis against several state-of-the-art methods, and advantages and limitations analysis. This comprehensive evaluation thoroughly demonstrates the effectiveness, practicality, rationality, and robustness of our proposed method.

The significance of this work lies not only in methodological innovation but also in bridging the gap between theoretical uncertainty modeling and practical industrial risk management. By providing a scalable, trustworthy, and interpretable FMEA framework, we empower organizations to make more informed, data-driven decisions in complex and uncertain environments, ultimately enhancing system safety and reliability.

The remainder of this paper is structured as follows. Section 2 reviews the preliminaries of FMEA, LGDM, and NCM. Section 3 details the data representation, conversion, and preprocessing strategy. Section 4 proposes our novel comprehensive weight determination method. The proposed NCM-LGDM-FMEA method is developed in Section 5. A practical case study is presented in Section 6. Result analysis, sensitivity analysis, both qualitative and quantitative comparative analysis, and advantages and limitations analysis are described in Section 7. Finally, Section 8 presents conclusions and future research directions.

## 2. Preliminaries

This section provides essential background on FMEA, LGDM, and the NCM to facilitate understanding of the proposed NCM-LGDM-FMEA method. It analyzes FMEA development, summarizes LGDM connotations, and describes NCM concepts and operations. Collectively, these subsections constitute a coherent preparatory foundation that logically connects existing knowledge with the integrated approach advanced in this study.

### 2.1. FMEA Models: A Critical Review

Contemporary FMEA research has extensively explored advancements aimed at enhancing its robustness and applicability. This review critically assesses leading contemporary techniques, with a specific focus on their approaches to uncertainty representation, expert weighting, LGDM, and data preprocessing. We highlight their inherent limitations in addressing these multi-faceted challenges.

Early enhancements often focused on representing the inherent fuzziness and randomness in expert evaluations. For instance, Li et al. [10] proposed an NCM-FMEA-2019 method where experts’ linguistic appraisals of RFs were transformed into NCMs. This approach allowed the calculated RPNs to retain uncertain information. However, this method was limited by its neglect of heterogeneous data, LGDM, and data preprocessing.

Liu et al. [19] built upon the NCM by integrating it with TOPSIS to develop the NCM-TOPSIS-FMEA-2019 method. Experts evaluate RFs using linguistic terms and then converted into NCMs for calculation. The weights of experts are determined based on their professional titles and work experience. However, their method did not take into account heterogeneous data, objective expert weights, LGDM, and data preprocessing.

Huang et al. [20] further explored uncertainty using Interval-Valued Intuitionistic Fuzzy Clouds (IVIFCs) to handle vagueness and randomness. Experts used linguistic terms to evaluate RFs and then converted into IVIFCs for calculation. Although expert weights combined subjective (experience, professional titles) and objective (deviation from known risk levels) components, a key limitation is the loss of uncertainty information when ranking values were converted to exact numbers. Furthermore, their method overlooked LGDM and data preprocessing, and objective weight calculation proved challenging.

Yu et al. [21] developed an FMEA model based on Data Envelopment Analysis (DEA) within LGDM. Multiple types of data, including exact numbers, interval numbers, and uncertain linguistic terms, can be employed to represent evaluations on different RFs. A notable weakness is the potential for different experts to use varying data types for the same RF. Furthermore, their method lacked data preprocessing, expert weighting, and sacrificed uncertainty in final exact numerical results.

Yu et al. [22] further integrated the NCM with an extended VIKOR method (NCM-VIKOR-FMEA-2021). Linguistic evaluations were processed by the NCM to address fuzziness and randomness. Expert weights incorporated subjective (professional position, service time) and objective (agreement, confidence) aspects. Still, the final ranking values were exact numbers, leading to uncertainty loss. Crucially, LGDM and data preprocessing remained unaddressed.

Subramanian et al. [23] introduced an FMEA using trapezoidal fuzzy numbers (TrFNs) and TOPSIS. Linguistic evaluations were converted to TrFNs, but final results were exact numbers, leading to uncertainty loss. This approach also did not consider LGDM or data preprocessing.

Chen et al. [24] presented a large-group FMEA using Relative Basic Uncertain Linguistic Information (RBULI) to manage opinion diversity. Experts were clustered using K-medoids. While input data incorporated uncertainty, derived O, S, D, and ranking values were exact numbers, resulting in information loss. Furthermore, their LGDM application involved a limited group size (20 experts) and lacked data preprocessing.

Sarwar et al. [15] constructed the RFIC-TOPSIS-FMEA-2023 method based on RFIC and TOPSIS to handle various uncertainties. Linguistic terms were converted to Triangular Fuzzy Numbers (TFNs) then NCMs. Although O, S, and D results preserved uncertainty via NCMs, final ranking values were exact, leading to information loss. This method also omitted LGDM, data preprocessing, and expert weighting.

Li et al. [25] developed a modified FMEA approach combining rough set and NCM theory. Linguistic terms from experts were converted to interval NCMs, preserving uncertainty in ranking. However, it did not account for LGDM, data preprocessing, or differential expert weights.

Zhang et al. [26] proposed an improved FMEA method based on multi-criteria group decision-making. Expert evaluations used Hesitant Fuzzy Sets (HFSs) converted to Normal Wiggly Hesitant Fuzzy Sets (NWHFSs). However, O, S, and D results were represented as NWHFSs, and final ranking values were exact numbers, causing uncertainty loss. Data preprocessing and expert weighting were also not addressed.

Wan et al. [27] proposed an FMEA model using Probabilistic Free Double Hierarchy Hesitant Linguistic Term Set (PFDHHLTS) under LGDM. While PFDHHLTS managed uncertainties, all evaluation results and final ranking values were exact numbers, leading to significant uncertainty loss. Furthermore, their LGDM method involved a limited number of experts and did not address data preprocessing or the assignment of different weights to different experts.

Mandal et al. [28] developed an FMEA model using Linguistic Z-Number (LZN) for medical science uncertainty. They proposed a consensus-driven approach for objective expert weights, criticizing pre-assigned weights. Yet, O, S, D, and final ranking values were exact, losing inherent uncertainty. LGDM and data preprocessing were not considered.

Zhang et al. [29] proposed a more recent NCM-based FMEA method (NCM-FMEA-2024) that effectively utilized heterogeneous data (exact numbers, interval numbers, linguistic terms, and linguistic expressions). Their method crucially calculated the RPN as an NCM, thereby preserving uncertainty throughout the process. Crucially, a common gap in the prior work was that it did not address LGDM, data preprocessing, or expert weighting.

While these advanced methods have demonstrably improved upon classical RPN-based FMEA, significant shortcomings persist, hindering their practical adoption in complex, large-scale industrial scenarios.

(1) Limited Handling of Heterogeneous Data: A near-universal limitation is the confinement to single evaluation data types or an inability to flexibly integrate mixed quantitative and qualitative data (e.g., exact numbers, interval numbers, NCMs, linguistic terms, and linguistic expressions) within a cohesive analytical framework for diverse FMs and RFs.

(2) Inadequate Uncertainty Propagation: Many methods attempt to represent uncertainty at the input or intermediate stages, but a pervasive issue is the loss of this uncertainty information when converting to exact numerical values for final ranking. This results in a loss of valuable information and reduced interpretability of the risk assessment.

(3) Weak or Absent Expert Weighting Mechanisms: While some studies incorporate expert weighting, few simultaneously integrate both subjective (e.g., experience, titles) and objective (e.g., data quality, consensus) factors comprehensively. The lack of sophisticated, dynamic weighting models often leads to biased or unreliable aggregation.

(4) Scalability Challenges in LGDM: The majority of FMEA studies, even those claiming to support group decisions, rely on a limited number of experts (typically 5–10). This is insufficient for complex modern systems where dozens or even hundreds of experts from diverse domains may be involved. A robust mechanism for scalable LGDM remains elusive.

(5) Absence of Data Preprocessing: The integrity of FMEA results is critically dependent on the quality of input data. Most existing methods overlook the essential step of robust data preprocessing to identify and handle invalid, inconsistent, or low-quality expert evaluations, which can severely distort risk prioritization.

The identified gaps collectively highlight the urgent need for a novel FMEA framework that can simultaneously address these interconnected challenges. Such a framework must enable the flexible handling of heterogeneous data, ensure end-to-end uncertainty propagation, implement dynamic and comprehensive expert weighting, support scalable LGDM, and incorporate robust data preprocessing.

### 2.2. Large-Scale Group Decision Making (LGDM)

With increasing complexity of modern industrial systems, FMEA may involve a large number of experts from diverse regions, departments, functions, and disciplines. Consequently, establishing large expert teams for identifying potential FMs and RFs has become essential. This broad participation highlights the critical need for an FMEA approach capable of effectively handling LGDM.

Traditionally, group decision making involved small expert groups (typically < 10). However, the evolution of information technology and societal demands (e.g., social networks, public participation, and e-democracy) has significantly increased participant numbers. LGDM now refers to collective decision making where a substantial number of participants contribute knowledge to solve complex problems. Unlike individual decision making, which is limited by a single perspective, LGDM leverages the combined expertise of a broad group to address intricate issues, thereby enhancing the decision-making process. The primary objective of LGDM is to synthesize diverse inputs into a coherent decision that accurately represents the collective judgment of the group [30]. While LGDM enhances diversity and robustness in risk assessments, effectiveness hinges not on the sheer number of experts but on input quality and coherence. Excessive participation may introduce noise and reduce consensus efficiency, especially in domain-specific contexts. To address this, our NCM-LGDM-FMEA framework dynamically preprocesses inputs and applies objective weighting to filter, prioritize, and aggregate expert judgments—ensuring reliable outcomes regardless of group size. The method is scalable (supporting dozens or hundreds of experts) yet equally effective in smaller teams, emphasizing intelligent, adaptive integration over maximal inclusion.

The application of LGDM offers several significant advantages, particularly for intricate or high-stakes decisions. Firstly, it facilitates the inclusion of a wide range of perspectives and expertise, substantially enhancing both the creativity and robustness of the final decision. This diversity of inputs can lead to innovative solutions that might not be conceived by smaller, more homogenous groups. Secondly, involving a large number of experts in the decision-making process effectively reduces the likelihood of errors or biases. Research on the “wisdom of the crowd” consistently demonstrates that collective decisions, especially when thoughtfully aggregated, often outperform individual judgments. Furthermore, the incorporation of various experts ensures that decisions are more representative of diverse interests and stakeholders. This inclusivity is crucial when decisions have broad implications across multiple sectors, organizations, or communities. Lastly, large groups can collectively assess risks, providing a more comprehensive understanding of potential hazards. By aggregating different risk perspectives, LGDM facilitates a more accurate and reliable risk management strategy, ultimately reducing the probability of overlooking significant threats.

Despite these substantial benefits, the integration of LGDM into FMEA remains a nascent area, with only a handful of studies successfully addressing its core challenges. Existing attempts often suffer from significant limitations. Liu et al. [30] proposed an FMEA method employing 20 experts using linguistic assessment information. However, their method lacked data preprocessing and the effective propagation of uncertain information. Additionally, experts were restricted to using a single linguistic informational format to represent evaluate results. Yu et al. [21] developed an LGDM-based FMEA method, also involving 20 experts, which utilized exact numbers, interval numbers, and uncertain linguistic terms. Nevertheless, their method did not address data preprocessing, expert weighting, or flexible expert participation. Moreover, representing final results with exact numbers sacrificed crucial uncertainty information. Chen et al. [24] presented a novel large-group FMEA model using RBULI to manage inherent ambiguity and vagueness in human decision making. Their LGDM approach, confined to 20 experts, similarly did not account for data preprocessing. Although input data incorporated uncertainty, the derived results were again exact numbers, leading to information loss. Wan et al. [27] proposed an FMEA model employing PFDHHLTS for uncertainty and fuzziness management in an LGDM environment with 22 experts. Yet, their evaluation results were all exact numbers, again resulting in uncertainty information loss. Furthermore, their LGDM method overlooked both data preprocessing and the assignment of differential expert weights.

Adapting traditional FMEA methods to LGDM under uncertainty thus presents significant challenges, making this a compelling and vital research area [31].

A primary limitation is the inherent diversity in expert input. While flexibility in format choice reduces experts’ cognitive load and error rates, it can also result in heterogeneous evaluations. Experts from various departments contribute decision information shaped by diverse cognitive abilities and experiences. A fixed expression format is insufficient for modern diversified decision environments, necessitating robust approaches for processing heterogeneous data, including exact numbers, interval numbers, NCMs, linguistic terms, and linguistic expressions. These heterogeneous data often contain various uncertainties, demanding effective processing and fusion methods to avoid information loss and distortion. This heterogeneity obstructs direct aggregation for collective assessment and consensus achievement. Therefore, managing heterogeneous information in LGDM is a significant concern [32]. Common approaches include [3]: (1) applying transformation functions for homogenization and subsequent aggregation, and (2) employing optimization models to derive collective assessments that align with individual inputs.

Effective LGDM requires weighting expert inputs by their data quality. However, many current methods either assume equal weights or rely on predefined values, overlooking critical quality differences. Such approaches can introduce subjectivity and limit the scientific rigor of the final decision, highlighting the need for dynamic, evidence-based weighting mechanisms. The diversity of expertise across domains leads to variations in knowledge, experience, and ability among experts. Consequently, their perceived importance, contribution, and impact may also differ. Assigning differential weights to experts is therefore a crucial topic in LGDM research.

Real-world contexts present a broader challenge: beyond heterogeneity and weighting, experts frequently exhibit complex uncertainties like fuzziness, randomness, and hesitation. However, most prior studies have only addressed a subset of these (e.g., fuzziness and hesitation) rather than concurrently modeling all three. Given its ability to comprehensively capture uncertainty through membership, non-membership, and hesitation degrees, the intuitionistic fuzzy set was employed as a decision-making tool to handle imprecise data in the quality evaluation of control systems [33]. More complex models, such as the interval rough integrated cloud model [34] and the dual-interval rough integrated cloud model [17] were introduced to cope with intrapersonal and interpersonal uncertainty, thereby addressing ambiguity and randomness simultaneously. Although the input data and intermediate calculations accounted for uncertainty, the final ranking outputs were reduced to exact numerical values, leading to a loss of information. Preserving and propagating these inherent uncertainties throughout the decision-making process is paramount.

Finally, a significant oversight in many previous LGDM methods is the lack of data preprocessing and validation. Even in LGDM with highly complex input data, such as uncertain linguistic terms [34] and multi-granularity linguistic environment [35], it is assumed that all experts can provide high-quality data for all indicators. Direct use of unfiltered expert inputs, especially in large groups where invalid or low-quality data are inevitable, can lead to erroneous outcomes. The increasing adoption of Web 2.0 technologies further compounds this challenge, as experts can dynamically join or leave the process, and decision environments constantly evolve with new information. This dynamic nature necessitates novel LGDM methods capable of adapting to evolving expert sets and continuously updated information landscapes [31].

In summary, despite the growing recognition of LGDM’s importance in modern FMEA applications, existing approaches still face critical limitations that hinder their practical utility. These include: (1) limited scalability beyond small-to-moderate-sized groups; (2) the inadequate handling of heterogeneous data formats across diverse experts; (3) insufficient mechanisms for dynamic, objective expert weighting based on data quality and consistency; (4) the absence of robust data preprocessing to detect and rectify invalid or outlier evaluations; and (5) failure to preserve uncertainty throughout the aggregation process. Collectively, these gaps underscore the urgent need for a next-generation LGDM framework that not only supports large-scale participation but also ensures data integrity, adaptability, fairness, and end-to-end uncertainty propagation—capabilities that are fundamental to reliable risk assessments in complex industrial systems.

### 2.3. Normal Cloud Model (NCM)

Based on fuzzy sets and probability theory, Li et al. [36] proposed a new cognition model known as the cloud model. This model simultaneously captures both the fuzziness and randomness of qualitative concepts. Among its variants, the Normal Cloud Model (NCM)—derived from the normal distribution and the Gaussian membership function—is the most widely adopted.

**Definition** **1** ([36])**.** *Let U represent the universe of discourse, and let T denote a qualitative concept within U. The qualitative concept T can be characterized by three numerical parameters: expectation (Ex), entropy (En), and hyper-entropy (He), where En ≥ 0 and He ≥ 0. If x∈U is a random instantiation of T, satisfying $x∼N(Ex,E{n^{\prime }}^{2})$ and $En^{\prime }∼N(En,He^{2})$, and if the certainty degree of x belonging to T satisfies:*

(1) $$\mu_{T}(x)=\exp\left(-\frac{{\left(x-Ex\right)}^{2}}{2{\left(En^{\prime }\right)}^{2}}\right),$$

*then the distribution of x within U is called an NCM, and each x associated with its certainty degree $\mu_{T}(x)$ is referred to as a cloud droplet of T.*

Consider two NCMs, ${\tilde{d}}_{1}$ = (*Ex*_1_, *En*_1_, *He*_1_) and ${\tilde{d}}_{2}$ = (*Ex*_2_, *En*_2_, *He*_2_), defined within the same universe of discourse, the arithmetic operation rules [29] are given as follows.

(2) $${\tilde{d}}_{1}⊕{\tilde{d}}_{2}=\left(Ex_{1}+Ex_{2},\sqrt{En_{1}^{2}+En_{2}^{2}},\sqrt{He_{1}^{2}+He_{2}^{2}}\right),$$

(3) $${\tilde{d}}_{1}⊖{\tilde{d}}_{2}=\left(Ex_{1}-Ex_{2},\sqrt{En_{1}^{2}+En_{2}^{2}},\sqrt{He_{1}^{2}+He_{2}^{2}}\right),$$

(4) $${\tilde{d}}_{1}⊗{\tilde{d}}_{2}=\left(Ex_{1}Ex_{2},\sqrt{{\left(En_{1}Ex_{2}\right)}^{2}+{\left(En_{2}Ex_{1}\right)}^{2}},\sqrt{{\left(He_{1}Ex_{2}\right)}^{2}+{\left(He_{2}Ex_{1}\right)}^{2}}\right),$$

(5) $${\tilde{d}}_{1}⊘{\tilde{d}}_{2}=\left(\frac{Ex_{1}}{Ex_{2}},\sqrt{{\left(\frac{En_{1}}{Ex_{2}}\right)}^{2}+{\left(\frac{Ex_{1}En_{2}}{Ex_{2}^{2}}\right)}^{2}},\sqrt{{\left(\frac{He_{1}}{Ex_{2}}\right)}^{2}+{\left(\frac{Ex_{1}He_{2}}{Ex_{2}^{2}}\right)}^{2}}\right).$$

The comparison rules are given as follows.

(6) $$\left\{\begin{array}{l} \mathrm{If}\;Ex_{1}>Ex_{2},\;\mathrm{then}\;{\tilde{d}}_{1}>{\tilde{d}}_{2}\\ \mathrm{If}\;Ex_{1}=Ex_{2}\;\mathrm{and}\;En_{1}<En_{2},\;\mathrm{then}\;{\tilde{d}}_{1}>{\tilde{d}}_{2}\\ \mathrm{If}\;Ex_{1}=Ex_{2},En_{1}=En_{2}\;\mathrm{and}\;He_{1}<He_{2},\;\mathrm{then}\;{\tilde{d}}_{1}>{\tilde{d}}_{2}\\ \mathrm{If}\;Ex_{1}=Ex_{2},En_{1}=En_{2}\;\mathrm{and}\;He_{1}=He_{2},\;\mathrm{then}\;{\tilde{d}}_{1}={\tilde{d}}_{2} \end{array}\right..$$

Consider *n* NCMs ${\tilde{d}}_{i}$ = (*Ex_i_*, *En_i_*, *He_i_*), *i* = 1, 2, …, *n*, defined within the same universe of discourse, and *w_i_* represents the weight of ${\tilde{d}}_{i}$. Yang et al. proposed two kinds of aggregation operators for NCMs [31]. One is the NCM synthetic operator *f_CS_*, and the other is the NCM weighted average operator *f_CWA_*.

(7) $$f_{CS}\left({\tilde{d}}_{1},{\tilde{d}}_{2},\ldots ,{\tilde{d}}_{n}\right)=\left(\frac{1}{n}\sum_{i=1}^{n}Ex_{i}\right.,\left.\frac{1}{6}\left(\underset{i}{\max}\left(Ex_{i}+3En_{i}\right)-\underset{j}{\min}\left(Ex_{j}-3En_{j}\right)\right),\sqrt{\sum_{i=1}^{n}He_{i}^{2}}\right),$$

(8) $$f_{CWA}\left({\tilde{d}}_{1},{\tilde{d}}_{2},\ldots ,{\tilde{d}}_{n};w_{1},w_{2},\ldots ,w_{n}\right)=\left(\overset{n}{\underset{i=1}{⊕}}\left(w_{i}⊗{\tilde{d}}_{i}\right)\right)⊘\left(\overset{n}{\underset{i=1}{⊕}}\left(w_{i}\right)\right).$$

According to the derivation by Yang et al. [31], if *w_i_* > 0 is an exact number for all *i* = 1, 2, …, *n*, and *w*_1_ + *w*_2_ + … + *w_n_* = 1, then the NCM weighted average operator in (8) simplifies to

(9) $$f_{CWA}\left({\tilde{d}}_{1},{\tilde{d}}_{2},\ldots ,{\tilde{d}}_{n};w_{1},w_{2},\ldots ,w_{n}\right)=\left(\sum_{i=1}^{n}w_{i}Ex_{i},\sqrt{\sum_{i=1}^{n}{\left(w_{i}En_{i}\right)}^{2}},\sqrt{\sum_{i=1}^{n}{\left(w_{i}He_{i}\right)}^{2}}\right).$$

## 3. Representation, Conversion, and Preprocessing of Heterogeneous Data

This section details the methods for heterogeneous data representation, conversion, and preprocessing.

### 3.1. Data Representation

In LGDM scenarios, FMEA experts may evaluate risks using heterogeneous data, including exact numbers, interval numbers, NCMs, linguistic terms, and linguistic expressions.

Assuming the universe of discourse U is scaled between 0 and 10 (U = [0, 10]), each FMEA expert provides evaluation data within this predefined scale.

An exact number is represented as *v*, where the evaluation result is exact, *d_i_* = *v*, with no associated uncertainty.

An interval number is represented as $I=[I^{L},I^{U}]$, where the evaluation result is a range, *d_i_* = *I*. This introduces deterministic uncertainty defined by the range $\left[I^{L},I^{U}\right]$.

An NCM is represented by three parameters *Ex*, *En* and *He*. The parameter *Ex* is the expectation that represents the most typical value of the subjective evaluation. The parameter *En* quantifies the uncertainty of the evaluation. Larger values indicate broader uncertainty ranges. The parameter *He* describes the uncertainty of *En*.

Inspired by Zadeh’s concept of linguistic variables and computing with words [37], subjective evaluations are more suitable to represent by linguistic terms. In this study, a linguistic term set including nine linguistic terms, ***H*** = {*none* (*n*), *very low* (*vl*), *low*, *slightly low* (*sl*), *medium* (*med*), *slightly high* (*sh*), *high*, *very high* (*vh*), *maximum* (*m*)}, is defined to represent the evaluation data on *O*, *S*, and *D*. A linguistic term typically embodies both fuzziness and randomness, making it suitable for representation using an NCM as $\tilde{d}$ (*Ex*, *En*, *He*).

In previous studies, a single linguistic term is generally used to express the evaluation result. Nevertheless, during the subjective evaluation, an FMEA expert may express hesitation between multiple linguistic terms or may prefer richer expressions, such as “between *medium* and *very high*” or “greater than *medium*”. The comparative linguistic expression modeled by context-free grammars *G_H_* [38] are much closer to human being, and thus can be used to elicit an FMEA expert’s evaluation data.

**Definition** **2** ([38])**.** *Suppose **H** = {t_1_, t_2_, …, t_n_} is the linguistic term set, then let G_H_ = (V_N_, V_T_, I, P), where:*

*V_N_ = {<primary term>, <composite term>, <unary relation>, <binary relation>, <conjunction>};*

*V_T_ = {lower than, greater than, at least, at most, between, and, t_1_, t_2_, …, t_n_};*

$I\in V_{N}$;

*P = {I ::= <primary term>|<composite term>,*

*<primary term> ::= T_0_|T_1_|…|T_n_,*

*<composite term> ::= <unary relation><primary term>| <binary relation><primary term><conjunction><primary term>,*

*<unary relation> ::= lower than|greater than|at least|at most,*

*<binary relation> ::= between,*

*<conjunction> ::= and}.*

This enables richer and more flexible linguistic representations, capturing the hesitations and nuanced preferences of experts.

### 3.2. Data Conversion

The heterogeneous data conversion methods are defined as the same as in [31]. The following methods convert an exact number *v*, an interval number $I=[I^{L},I^{U}]$, a linguistic term *t*, or a linguistic expression *L_t_* into its corresponding NCM.

(10) $$v\to \tilde{d}(v,0,0),$$

(11) $$I\to \tilde{d}\left(\frac{I^{L}+I^{U}}{2},\frac{I^{U}-I^{L}}{6},0\right),$$

(12) $$t\overset{f_{encoded}}{\to }\tilde{d}(Ex,En,He),$$

(13) $$L_{t}\overset{f_{G_{H}}}{\to }HCLTS\overset{f_{CS}}{\to }\tilde{d}(Ex,En,He).$$

In (12), the *f_encoded_* method represents the encoded method based on fuzzy statistics and membership function fitting [39], including several processes such as data collection, data preprocessing, fuzzy statistics, membership function fitting, and parameter mapping. Yang et al. [39] encoded 32 linguistic terms into their corresponding NCMs using a dataset collected from 175 individuals on the universe of discourse [0, 10]. Based on the resulting codebook, nine linguistic terms are introduced for credibility assessments [40]. The use of a standardized codebook ensures accuracy and consistency in representing linguistic terms for decision-making applications like FMEA. In this work, we directly adopt the nine NCMs for linguistic terms as listed in Table 1. This approach avoids subjective parameter setting and ensures that these linguistic terms are empirically validated.

If an FMEA expert selects one of these linguistic terms to express the evaluation result, then the linguistic term is mapped to its corresponding NCM using Table 1.

To deal with linguistic expressions, Huang and Yang [41] introduced the concept of Hesitant Cloud Linguistic Term Sets (HCLTSs).

**Definition** **3** ([41])**.** *Let $H=\{T_{i}\left|i=0,1,\ldots ,2\tau ,\;\tau \in N^{*}\right.\}$ be a finite and totally ordered discrete linguistic term set, where T_i_ is a linguistic term modeled by an NCM ${\tilde{d}}_{i}$ = (Ex_i_, En_i_, He_i_). An HCLTS H_S_ is defined as an ordered finite subset of the consecutive linguistic terms in H.*

An FMEA expert may provide the evaluation data using a linguistic expression. Let the linguistic term set *H* be defined as described in Table 1. The context-free grammars *G_H_* described in *Definition 2* are used to generate linguistic expressions. First, each linguistic expression is transformed into an HCLTS by the following transformation function $f_{G_{H}}$.

(14) $$\left\{\begin{array}{l} f_{G_{H}}(T_{i})=\{T_{i}|T_{i}\in H\}\\ f_{G_{H}}(\mathrm{at}\;\mathrm{most}\;T_{i})=\{T_{j}|T_{j}\in H\;\mathrm{and}\;T_{j}\leq T_{i}\}\\ f_{G_{H}}(\mathrm{lower}\;\mathrm{than}\;T_{i})=\{T_{j}|T_{j}\in H\;\mathrm{and}\;T_{j}<T_{i}\}\\ f_{G_{H}}(\mathrm{at}\;\mathrm{least}\;T_{i})=\{T_{j}|T_{j}\in H\;\mathrm{and}\;T_{j}\geq T_{i}\}\\ f_{G_{H}}(\mathrm{greater}\;\mathrm{than}\;T_{i})=\{T_{j}|T_{j}\in H\;\mathrm{and}\;T_{j}>T_{i}\}\\ f_{G_{H}}(\mathrm{between}\;T_{i}\;\mathrm{and}\;T_{j})=\{T_{k}|T_{k}\in H\;\mathrm{and}\;T_{i}\leq T_{k}\leq T_{j}\} \end{array}\right..$$

Then, the HCLTS is converted into an NCM using the NCM synthetic operator *f_CS_*, as given by (7).

The above methods ensure that heterogeneous data types, including numeric and linguistic forms, can be converted into NCMs. These transformations ensure that data from different FMEA experts, regardless of the format in which it was originally expressed, can be used consistently in the overall risk evaluation framework. Thus, various uncertainties inherent in subjective human evaluations are preserved and propagated throughout subsequent decision-making processes. This process preserves uncertainties and facilitates unified analysis in subsequent computations.

### 3.3. Data Preprocessing

In LGDM scenarios, data preprocessing is crucial for ensuring the quality and consistency of the data collected. The preprocessing process aims to eliminate unreasonable or erroneous data that could distort the decision-making process. In [31], an effective data preprocessing approach is presented. Suppose the dataset *D* = {*d_i_*, *i* = 1, …, *n*} have been collected from *n* experts, where each *d_i_* represents the data provided by the *i*-th expert, the data preprocessing consists of four steps: invalid data filtering, bad data processing, outlier processing, and tolerance limit processing.

*Step 1. Invalid data filtering:* During the data collection phase, some experts may provide data that fall outside the universe of discourse, making them invalid for further processing. Let *V*, *I*, ${\tilde{d}}_{i}$, *T*, and *L* represent exact numbers, interval numbers, NCMs, linguistic terms and linguistic expressions, respectively. The data *d_i_* that does not satisfy the following conditions is deemed invalid and is discarded.

(15) $$\left\{\begin{array}{l} 0\leq d_{i}\leq 10,\forall d_{i}\in V\\ 0\leq d_{i}^{L}\leq 10,0\leq d_{i}^{U}\leq 10,\mathrm{and}\;d_{i}^{L}\leq d_{i}^{U},\forall d_{i}\in I\\ 0\leq d_{i}(Ex)\leq 10,d_{i}(En)\geq 0,\mathrm{and}\;d_{i}(He)\geq 0,\forall d_{i}\in \tilde{d}\\ d_{i}\in H,\forall d_{i}\in T\\ d_{i}\in G_{H},\forall d_{i}\in L \end{array}\right..$$

After invalid data is filtered out, there are *n*_1_ ≤ *n* data left.

The remaining *n*_1_ data are then converted into NCMs as *D*’ = {${\tilde{d}}_{i}$ = (*Ex_i_*, *En_i_*, *He_i_*), *i* = 1, …, *n*_1_}, by the methods described in Section 3.2.

*Step 2. Bad data processing:* This step addresses low-quality data that exhibit unreasonable levels of uncertainty. Such data are likely to contain excessive noise or other issues that make them unreliable. To filter out bad data, the following condition must be satisfied.

(16) $$\left\{\begin{array}{l} 3(En_{i}+3He_{i})\leq 10\\ 3He_{i}\leq En_{i} \end{array}\right..$$

The condition ensures that only data with reasonable uncertainties are kept. After this step, the remaining dataset will contain *n*_2_ ≤ *n*_1_ data items.

*Step 3. Outlier processing:* This step eliminates outliers using the Box and Whisker test, which identifies data points that fall outside the expected range based on the interquartile range (IQR). The data ${\tilde{d}}_{i}$ that does not satisfy the following conditions are considered outliers and discarded.

(17) $$\left\{\begin{array}{l} Q_{Ex}(0.25)-1.5IQR_{Ex}\leq Ex_{i}\leq Q_{Ex}(0.75)+1.5IQR_{Ex}\\ \begin{array}{l} 0\leq En_{i}\leq Q_{En}(0.75)+1.5IQR_{En}\\ 0\leq He_{i}\leq Q_{He}(0.75)+1.5IQR_{He} \end{array} \end{array}\right..$$

where *Q*(0.25) and *Q*(0.75) are the first and third quartiles of the data, respectively, and *IQR* = *Q*(0.75) − *Q*(0.25) is the interquartile range between the first and third quartiles.

This ensures that only data within a reasonable range (based on the distribution of the data) remain. After this step, the dataset will contain *n*_3_ ≤ *n*_2_ data items.

*Step 4. Tolerance limit processing:* This step ensures that the data is within a tolerable range. The means *m_Ex_*, *m_En_*, and *m_He_* and standard deviations *σ_Ex_*, *σ_En_*, and *σ_He_* are calculated, based on all the remaining data items {${\tilde{d}}_{i}$ = (*Ex_i_*, *En_i_*, *He_i_*), *i* = 1, …, *n*_3_}. Only the data ${\tilde{d}}_{i}$ satisfying the following conditions will be kept

(18) $$\left\{\begin{array}{l} m_{Ex}-k\sigma_{Ex}\leq Ex_{i}\leq m_{Ex}+k\sigma_{Ex}\\ \begin{array}{l} 0\leq En_{i}\leq m_{En}+k\sigma_{En}\\ 0\leq He_{i}\leq m_{He}+k\sigma_{He} \end{array} \end{array}\right..$$

where *k* is related to data size to ensure that 90% data (*alpha* = 0.1) will be left with 95% confidence (*γ* = 0.05).

The data points that deviate excessively from these tolerance limits are considered abnormal and are discarded. After this step, there are *n*_4_ ≤ *n*_3_ remaining data items finally.

These preprocessing steps ensure that the dataset used for FMEA is valid, reliable, and free of outliers or anomalies, enabling more accurate decision making.

## 4. Comprehensive Weights Determination Method

Previous group decision-making methods rarely assigned weights according to both the quality of evaluation data and the diverse professional background of FMEA experts. In LGDM scenarios, it is essential to quantitatively represent and assess the quality of evaluation data, while also assigning appropriate weights to experts based on their professional titles and experience. This section presents a novel approach for determining comprehensive weights.

### 4.1. Data Weights

The *UD* measures the fuzziness and randomness of evaluation data. The *DD* is defined based on Theil’s inequality coefficient (TIC) to reflect the degree of difference between individual and group evaluations. Then, both *UD* and *DD* are used to measure the quality of the evaluation data quantitatively. The data weight is calculated by a two-dimensional Gaussian function of *UD* and *DD*.

#### 4.1.1. Uncertainty Degree

The *UD* is computed to reflect the uncertainty level of the evaluation data quantitatively. An NCM represents both fuzziness and randomness simultaneously by two parameters *En* and *He*. A larger *En* means a wider coverage by the concept. The parameter *He* is the entropy of *En*, which describes the uncertainty of *En*. Thus, the *UD* of the evaluation data can be calculated by the two parameters *En* and *He* of its corresponding NCM [31].

Let the evaluation data of an FMEA expert *e_i_* is represented by or converted into an NCM as ${\tilde{d}}_{i}$ = (*Ex_i_*, *En_i_*, *He_i_*). The *UD* of *e_i_* is calculated as

(19) $$UD_{i}=3\times (En_{i}+3\times He_{i})/10.$$

The invalid data filtering and bad data processing steps described in Section 3.2 ensure that 0 ≤ *UD* ≤ 1. A smaller *UD* is preferable, indicating fewer uncertainties.

**Remark** **1.** *For each FMEA expert, the UDs of the evaluation data on O, S, and D are calculated separately. Therefore, the three UDs of the same expert e_i_ may be different across O, S, and D.*

#### 4.1.2. Difference Degree

As FMEA expert may have different cognitive abilities, professional levels, and knowledge backgrounds, their evaluation results are often not identical. To consider the degree of consensus in group decision making, the quantitative analysis of *DD* may help to further improve accuracy and efficiency in LGDM processes. The *DD* quantifies the difference between individual and group evaluation data. Smaller *DD* means stronger consistency between individual and group evaluations, and thus a greater weight should be given.

TIC developed by Theil has been used widely in the field of simulation model validation [40]. TIC is a measure between 0 and 1, reflecting the degree of difference between two vectors. All the three parameters of an NCM are used to calculate the difference quantitatively. An NCM can be regarded as a three-dimensional vector. Thus, the difference between two NCMs ${\tilde{d}}_{i}$ and ${\tilde{d}}_{j}$ is calculated as

(20) $$D_{ij}=\frac{{\left\|{\tilde{d}}_{i}-{\tilde{d}}_{j}\right\|}_{2}}{{\left\|{\tilde{d}}_{i}\right\|}_{2}+{\left\|{\tilde{d}}_{j}\right\|}_{2}}.$$

Suppose that there are *n* experts *E* = {*e*_1_, *e*_2_, …, *e_n_*} in the subgroup, the *DD* of *e_i_* is calculated as

(21) $$DD_{i}=\frac{1}{n}\sum_{j=1}^{n}D_{ij}.$$

It is evident that 0 ≤ *DD* ≤ 1. A lower *DD* indicates a stronger alignment with others and thus a higher weight.

**Remark** **2.** *For each FMEA expert, the DDs of the evaluation data on O, S, and D are calculated separately. Therefore, the three DDs of the same expert e_i_ may be different across O, S, and D.*

#### 4.1.3. Two-Dimensional Gaussian Weighting Model

Both *UD* and *DD* are proposed to measure the quality of the evaluation data quantitatively from different perspectives. The weight decreases with the increase in *UD* and *DD*. In order to integrate *UD* and *DD*, a two-dimensional Gaussian function is presented to calculate data weights for each expert *e_i_* across *O*, *S*, and *D, respectively.*

(22) $$w_{i}^{\mathrm{data}}=e^{-\frac{\left({\left(UD_{i}/\bar{UD}\right)}^{2}+{\left(DD_{i}/\bar{DD}\right)}^{2}\right)}{2}}.$$

where $\bar{UD}$ and DD are the mean values of *UDs* and *DDs* of experts, respectively.

It is evident that 0 ≤ $w_{i}^{\mathrm{data}}$ ≤ 1. Smaller *UD* and *DD* mean better data quality and thus lead to higher weights.

**Remark** **3.** *As the UD and DD of the evaluation data of an FMEA expert on O, S, and D are calculated separately, the data weights of the same expert e_i_ may be different across O, S, and D.*

**Example** **1.** *Consider three NCMs (7.1050, 1.0270, 0.0410), (5.9973, 2.0793, 0.0871), and (3.1290, 1.7208, 0.0734). According to (19), their UDs are calculated as 0.3450, 0.7022, and 0.5823, respectively. Based on (21), their DDs are obtained as 0.0929, 0.0610, and 0.1370, respectively. Subsequently, using (22), the data weights assigned to the three NCMs are calculated as 0.5163, 0.3558, and 0.2076, respectively.*

### 4.2. Identity Weights

Considering that the professional title and work experience represent the level of competence to a certain extent, the FMEA experts are divided into four identity categories.

Suppose there are *n* FMEA experts in the LGDM process. These experts are divided into four subgroups as G_1_, G_2_, G_3_ and G_4_ according to their background including professional titles and work experience in the FMEA field, as described in Table 2.

More work experience and higher professional title are preferred, so the identity weight of expert *e_i_* from these subgroups should satisfy $w_{G_{1}}>w_{G_{2}}>w_{G_{3}}>w_{G_{4}}$. The identity weights are defined as

(23) $$w_{i}^{\mathrm{identity}}=\left\{\begin{array}{ll} \alpha & e_{i}\in G_{1}\\ 0.8\times \alpha & e_{i}\in G_{2}\\ 0.7\times \alpha & e_{i}\in G_{3}\\ 0.5\times \alpha & e_{i}\in G_{4} \end{array}\right..$$

where 0 ≤ *α* ≤ 1 is a trade-off coefficient that adjusts the data weights and identity weights. A higher *α* places greater importance on identity weights—reflecting the influence of expert role—while a lower *α* prioritizes data weights derived from objective information. In this study, *α* = 0.5 is adopted to achieve a balanced integration of both expert role and data characteristics, ensuring neither component dominates the final risk assessment.

**Remark** **4.** *For the same FMEA expert, his/her identity weights are identical across O, S, and D.*

### 4.3. Comprehensive Weights

The final weight *w_i_* for expert *e_i_* on *O*, *S*, or *D* is determined comprehensively by combining the data weight and the identity weight.

(24) $$w_{i}=\frac{w_{i}^{\mathrm{data}}+w_{i}^{\mathrm{identity}}}{\sum_{i=1}^{n}\left(w_{i}^{\mathrm{data}}+w_{i}^{\mathrm{identity}}\right)}.$$

**Remark** **5.** *Separate comprehensive weights are calculated across O, S, and D, to ensure a tailored representation of each expert’s contributions across different aspects of FMEA evaluations.*

This method combines data quality measures (*UD* and *DD*) with individual competence (identity weights) to assign comprehensive weights. By reflecting both data reliability and the expertise of experts, the approach improves the accuracy and fairness of LGDM in FMEA.

## 5. The Proposed FMEA Method

**Figure 1:**
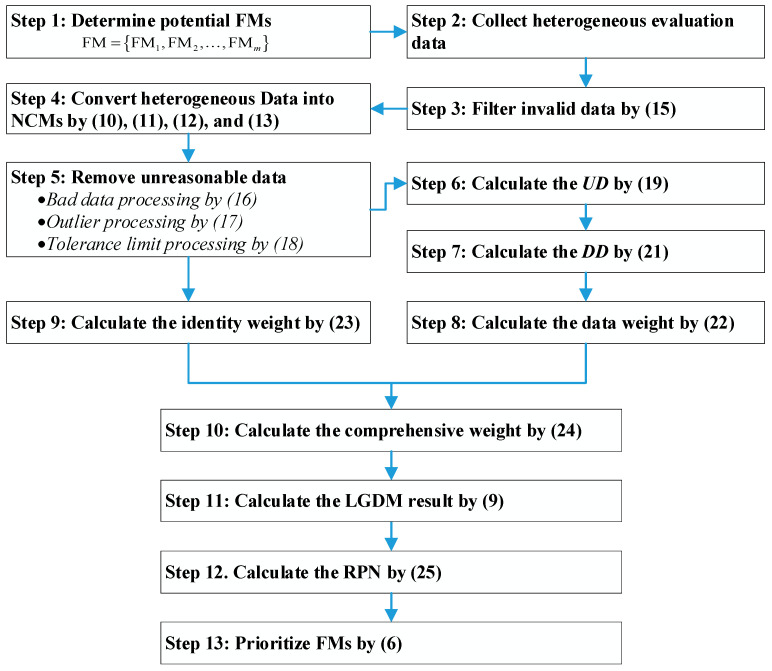
The framework of the proposed NCM-LGDM-FMEA method.

In this section, a novel FMEA method is developed based on LGDM and NCM. This NCM-LGDM-FMEA method is capable of processing diverse types of evaluation data, including exact numbers, interval numbers, NCMs, linguistic terms, and linguistic expressions. The framework of the proposed approach is illustrated in Figure 1, which consists of the following thirteen steps.

**Step 1: Determine potential FMs.**

Based on historical data and domain experience, the FMEA management team determines a set of potential FMs to be analyzed, denoted as **FM** = {FM*_i_*, *i* = 1, 2, …, *m*}. Each FM is evaluated against three RFs: O, S, and D.

**Step 2: Collect heterogeneous evaluation data.**

For each RF of each FM, multiple experts contribute evaluations under the LGDM environment. Expert participation is tailored according to individual expertise; different experts may evaluate different subsets of RFs and FMs. For instance, the evaluation of O for FM_1_ may involve experts Zhang, Li, Wang, Yang, Liu, Zhao, Sun, and Qian et al., while the evaluation of D for FM_1_ may involve Li, Yang, Wu, He, Ma and Lin et al. Evaluations can be collected via online platforms, in-person meetings, or written correspondence.

Due to individual preferences, varying levels of understanding of the issues, and the inherent vagueness, randomness, and hesitation in human cognition, different experts in LGDM may utilize different types of data to represent their evaluation information. In order to enhance the flexibility, diversity, and accuracy of opinions, every expert is allowed to express the evaluation information on an RF of an FM using any type of data. Thus, the evaluation data on each RF of each FM collected from *n* experts {*e_k_*, *k* = 1, 2, …, *n*} can be denoted as a dataset **D** = {*d_k_*, *k* = 1, …, *n*}, where *d_k_* from *e_k_* may be an exact number, an interval number, an NCM, a linguistic term or a linguistic expression. This approach fosters different perspectives and cognitive styles, enabling a richer and more accurate representation of evaluation information, which can ultimately lead to more informed decision making. This variability can lead to a more comprehensive understanding of the situation, but it also requires robust aggregation and analysis techniques to achieve coherent results.

**Remark** **6.** *Experts should be selected from varied professional backgrounds with relevant skills and experience. The composition and number n of experts may differ across RFs and FMs. Each expert may evaluate one or more (may be not all) RFs and FMs.*

**Step 3: Filter invalid data.**

Evaluations that fall outside the predefined universe of discourse are identified via (15) and discarded. The remaining *n*_1_ (≤*n*) valid entries proceed to subsequent steps.

**Step 4: Convert heterogeneous data into NCMs.**

Remaining valid data are converted into corresponding NCM representations using methods outlined in Section 3.2. Therefore, the dataset *D* are converted into its corresponding NCM dataset $\tilde{D}=\{{\tilde{d}}_{k},k=1,2,\ldots ,n_{1}\}$, where ${\tilde{d}}_{k}$ denotes the NCM for each valid entry.

**Step 5: Remove unreasonable data.**

Unreasonable data are eliminated through successive processing using (16), (17), and (18). After these operations, *n*_4_ ≤ *n*_1_ data items remain for subsequent calculations.

**Step 6: Calculate the *UD*.**

The *UD* of an NCM is calculated by (19).

**Step 7: Calculate the *DD*.**

The *DD*, quantifying the consistency between individual and group evaluations, is calculated by (21).

**Step 8: Calculate the data weight.**

Data weights are computed using a two-dimensional Gaussian function as per (22).

**Step 9: Calculate the identity weight.**

The identity weight of an expert, reflecting professional title and work experience, is calculated by (23).

**Step 10: Calculate the comprehensive weight.**

The comprehensive weight *w_k_* for expert *e_k_* is obtained by integrating the data-driven weight and the identity-based weight according to (24).

**Step 11: Calculate the LGDM result.**

The LGDM result is calculated using the NCM weighted average operator described in (9) to aggregate data ${\tilde{d}}_{k}$ with weight *w_k_*.

**Step 12: Calculate the RPN.**

Perform steps 2 to 11 on O, S, and D, respectively, to obtain the LGDM results $\tilde{O}$, $\tilde{S}$, and $\tilde{D}$.

The $\tilde{RPN}$ of each FM is calculated by multiplying the LGDM results for $\tilde{O}$, $\tilde{S}$, and $\tilde{D}$.

(25) $$\tilde{RPN}=\tilde{O}⊗\tilde{S}⊗\tilde{D}.$$

When the $\tilde{RPN}$ is higher, it means the problem is more serious and needs to be fixed quickly. This also indicates that there is a greater chance of causing harm, so we need to take stronger and more careful actions to reduce the risks effectively. The $\tilde{RPN}$ is also represented by an NCM, as uncertainties in O, S, and D propagate to the RPN. If O, S, and D possess uncertainties, the calculated RPN will retain their uncertainties.

**Step 13: Prioritize FMs.**

All FMs are prioritized based on their RPNs, following the comparison rules of NCMs outlined in (6). FMs with higher ranks should be addressed as a priority.

The algorithm for the proposed NCM-LGDM-FMEA method is summarized in Algorithm 1, providing an executable workflow for practical implementation.
**Algorithm 1:** The proposed NCM-LGDM-FMEA 1: Determine **FM** = {FM*_i_*, *i* = 1, 2, …, *m*} 2: **for** each ${\mathrm{FM}}_{i}\in FM$ **do** 3:  **for** each RF∈{O,S,D} **do** 4:   Collect heterogeneous evaluation data **D** = {*d_k_*, *k* = 1, …, *n*}. 5:   Filter invalid data by (15), $D\to D^{\prime }$. 6:   **for** each $d_{k}\in D^{\prime }$ **do** 7:    Convert *d_k_* into an NCM ${\tilde{d}}_{k}$ as Section 3.2, $D^{\prime }\to \tilde{D}$. 8:    Bad data processing on ${\tilde{d}}_{k}$ by (16), $\tilde{D}\to {\tilde{D}}^{\prime }$. 9:    Outlier processing on ${\tilde{d}}_{k}$ by (17), ${\tilde{D}}^{\prime }\to {\tilde{D}}^{\prime \prime }$. 10:    Tolerance limit processing on ${\tilde{d}}_{k}$ by (18), ${\tilde{D}}^{\prime \prime }\to {\tilde{D}}^{\prime \prime \prime }$. 11:   **end for** 12:   **for** each ${\tilde{d}}_{k}\in {\tilde{D}}^{\prime \prime \prime }$ **do** 13:    Calculate the *UD_k_* of ${\tilde{d}}_{k}$ by (19). 14:    Calculate the *DD_k_* of ${\tilde{d}}_{k}$ by (21). 15:    Calculate the data weight $w_{k}^{\mathrm{data}}$ of ${\tilde{d}}_{k}$ by (22). 16:    Calculate the identity weight $w_{k}^{\mathrm{identity}}$ of *e_k_* by (23). 17:    Calculate the comprehensive weight *w_k_* of *e_k_* by (24). 18:    Calculate the LGDM result by (9) to aggregate ${\tilde{d}}_{k}$ with *w_k_*. 19:   **end for** 20:   Obtain $\tilde{O}$, $\tilde{S}$, and respectively. 21:  **end for** 22:  Calculate ${\tilde{RPN}}_{i}$ of FM*_i_* by (25). 23: **end for** 24: Prioritize {FM*_i_*} by (6) based on their RPNs.

**Remark** **7.** *While some prior improved FMEA methods have accommodated uncertain input information, including fuzzy sets and linguistic terms, they fail to quantify the extent of uncertainties present in the RPN result. This approach explicitly quantifies uncertainties in $\tilde{RPN}$ results, addressing a critical limitation of earlier works. This distinction underscores the primary difference between our approach and previous methods.*

The runtime of the proposed NCM-LGDM-FMEA algorithm is primarily determined by three key parameters: the number of FMs denoted as *m*, the number of RFs (O, S, and D), and the number of experts involved in the evaluation, denoted as *n*. The core operations include processing each FM, evaluating it across all RFs, and aggregating expert evaluations. Consequently, the baseline computational complexity scales as *O*(*m*) × *O*(3) × *O*(*n*), which reduces to *O*(*m* × *n*), since the number of RFs is a small constant. However, a more refined analysis reveals that the computation of objective expert weights involves calculating pairwise *DD* among all experts. This step necessitates a comparison between every pair of experts, resulting in a computational burden of *O*(*n*^2^). As this operation is performed for each FM and RF, it significantly impacts the overall complexity. Thus, the total computational complexity of the proposed algorithm can be expressed as *O*(3 × *m* × *n*^2^). In practical FMEA applications, the number of FMs *m* is relatively small (e.g., 3 to 10). However, in LGDM FMEA applications for complex systems, the number of experts *n* can be large due to multidisciplinary involvement. Despite this, the algorithm exhibits favorable scalability: its complexity grows quadratically with respect to the number of experts, but remains linear in the number of FMs. This makes the algorithm efficient and well suited for real-world implementation, particularly when expert consensus is critical and the number of experts is moderate. This theoretical complexity analysis confirms that the proposed NCM-LGDM-FMEA algorithm maintains both computational efficiency and practical feasibility in diverse FMEA contexts.

## 6. Case Study

This section demonstrates the practical implementation and validates the effectiveness and feasibility of the proposed NCM-LGDM-FMEA method through a real-world case study: the air data system (ADS) of modern airplanes.

The ADS in modern aircraft, such as the Boeing 737, is paramount for providing accurate and reliable information regarding the aircraft’s speed, altitude, and other essential flight parameters. It processes environmental data, including total and static air pressure, as well as temperature, which directly influences the aircraft’s navigation, flight control, and overall safety.

**Figure 2:**
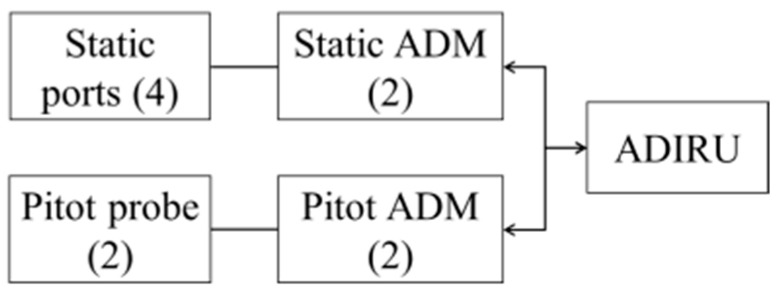
Schematic diagram of the air data system.

As shown in Figure 2, the ADS comprises several key components: the total and static pressure system, the Air Data Module (ADM), and the Air Data and Inertial Reference Unit (ADIRU). The total and static pressure system acquires air pressure inputs from three pitot probes and six static ports strategically located on the aircraft fuselage. Static air pressure represents the ambient atmospheric pressure around the aircraft, while pitot air pressure is measured at the pitot probe tube as a direct consequence of the aircraft’s forward motion. The ADM is a critical component responsible for sensing these air pressures from the pitot tubes and static ports. It converts the analog air pressures into electrical signals, which are then transmitted to the ADIRU via ARINC 429 data buses. The ADIRU itself is typically composed of two main parts: the Air Data Reference (ADR) and the Inertial Reference (IR). The ADR specifically utilizes the pressure data received from the ADM to calculate crucial flight parameters such as airspeed and barometric altitude, which are fundamental inputs for the autopilot and navigation systems.

Failures within the ADS can lead to severe consequences, significantly impacting aircraft performance, safety, and control. Any malfunction in the ADS can result in erroneous information being supplied to the flight crew and autopilot systems, potentially instigating a cascade of critical issues. In Air France Flight 447 (2009), the aircraft’s pitot tubes became obstructed by ice crystals, leading to inconsistent airspeed readings. This contributed to profound confusion among the flight crew, who, misinterpreting the faulty airspeed information, were unable to take appropriate corrective actions, ultimately resulting in the loss of the aircraft. American Airlines Flight 191 (1979) involved a loss of the ADS, where the aircraft’s airspeed and altitude information became inconsistent due to a failure in one of the Air Data Computers (ADCs). This rendered it exceptionally difficult for the flight crew to maintain safe flight parameters. These incidents highlight the urgent need for robust failure prevention and meticulous maintenance planning for the ADS.

Given the inherent complexity and interdependency of the ADS, decisions regarding failure prioritization and maintenance planning necessitate collaborative input from multiple domain experts. This includes, but is not limited to, avionics engineers, flight safety officers, and maintenance technicians. The proposed framework’s integration of LGDM under uncertainty is particularly well suited for this context for several reasons. (1) LGDM ensures that diverse experts provide invaluable insights on potential FMs based on their unique perspectives and experience. This multi-angled analysis substantially reduces the risk of overlooking critical FMs. (2) By synthesizing expert opinions from multiple disciplines, LGDM facilitates the formation of a more comprehensive and robust consensus on the severity and likelihood of various FMs. This aids in identifying the FMs with the potentially most severe consequences. (3) The ADS operates under dynamically varying conditions, where environmental factors such as temperature, pressure, and airspeed constantly change during flight, inevitably introducing measurement variability and uncertainty. The NCM leveraged by our method is ideally poised to model and manage these inherent uncertainties, providing a more realistic assessment of risk.

To assess the potential risks associated with ADS failures, an expert team systematically evaluated different modules of the system to identify typical FMs and their potential effects. Five representative FMs were identified based on the common causes of ADS malfunction as listed in Table 3.

For the evaluation of FM_1_, a total of 50, 52, and 49 experts provided various types of evaluation data for O, S, and D, respectively. These experts were divided into four subgroups (G_1_, G_2_, G_3_, G_4_). Specifically, for the evaluation of O, the 50 experts were distributed as 14, 12, 12, and 12 experts from G_1_, G_2_, G_3_, and G_4_, respectively. For S, the 52 experts comprised 15, 13, 12, and 12 experts from G_1_, G_2_, G_3_, and G_4_. Lastly, for D, the 49 experts were composed of 14, 12, 12, and 11 experts from G_1_, G_2_, G_3_, and G_4_.

Each expert was permitted to express their evaluation result for each RF of each FM using a variety of formats, including exact numbers, interval numbers, NCMs, linguistic terms, or linguistic expressions. The raw data for FM_1_ from all experts are provided in Table 4.

Invalid data outside the universe of discourse were filtered out by (15). The remaining valid data were then converted into their corresponding NCM representations as listed in Table 5, using the methods described in Section 3.2. It is important to note that some invalid data, which were outside the predefined universe of discourse, were removed at this stage, resulting in no corresponding NCMs being generated for them.

Subsequently, unreasonable data were eliminated using (16), (17), and (18). After these operations, 43, 40 and 44 data items remained for O, S, and D, respectively.

For each remaining NCM, the *UD* and *DD* were calculated by (19) and (21). The data weights and identity weights (with *α* = 0.5) were then calculated by (22) and (23), respectively. Consequently, the comprehensive weights were derived using Equation (24). It should be noted that both data weights and comprehensive weights were not calculated for those data points that were identified as removed or unreasonable during the earlier preprocessing steps, ensuring that only reliable expert input contributed to the final aggregation. The calculated data weights and comprehensive weights are presented in Table 6.

Then, LGDM results were calculated using the NCM weighted average operator described in (9) to form the group decision-making results. LGDM results for O, S, and D are (4.8184,0.1558,0.0065), (6.6190,0.1356,0.0053), and (4.0780,0.1601,0.0065), respectively.

Lastly, the $\tilde{RPN}$ value of FM_1_ was calculated as (130.0587,7.1313,0.2900) by multiplying O, S, and D. The $\tilde{RPN}$ values of other FMs were calculated according to the same process (please refer to the Supplementary Materials for the complete dataset).

The ranking was determined following the comparison rules of NCMs outlined in (6). The results are presented in Table 7.

As listed in Table 7, the risk priority of the five FMs is ${\mathrm{FM}}_{3}≻{\mathrm{FM}}_{1}≻{\mathrm{FM}}_{5}≻{\mathrm{FM}}_{2}≻{\mathrm{FM}}_{4}$. Based on the ranking outcomes of the five FMs derived by the proposed method, the project team can more effectively identify improvement needs and implement enhanced control and supervision strategies for FMs associated with higher severity. Simultaneously, targeted control measures are applied to less critical FMs to optimize overall project reliability while considering cost-effectiveness. For instance, FM_3_ emerges as the highest-priority risk. Consequently, it is imperative for the project’s safety management to prioritize proactive interventions, emphasizing both preventive measures and detection controls to mitigate potential risks associated with FM_3_. Thus, FM_3_ is most critical and should be given top priority for prevention and control measures.

Additionally, it can be seen that the $\tilde{RPN}$ value represented by an NCM can reflect the degree of uncertainty through two parameters, *En* and *He*. The uncertainty arises from both subjective and objective factors when evaluating O, S, and D of the FM. If there is uncertainty in O, S, and D, it should be preserved in the RPN result. Although some previous FMEA methods could accept uncertain input information, such as fuzzy sets and linguistic terms, they were unable to indicate the extent of that uncertainty in the final RPN result, and the outcome is an exact number. Nevertheless, our results can offer richer insights. Therefore, it can be concluded that a more accurate and effective risk ranking of FMs can be achieved using our proposed method. The complete NCM representations (including *Ex*, *En*, and *He*) are retained throughout the analysis, enabling decision makers to apply their own judgment and preference when interpreting the results. For instance, if a decision maker places greater emphasis on risk aversion or stability, they may choose to prioritize alternatives with lower *En* and *He*—i.e., those with more precise and reliable assessments—even at the cost of slightly lower *Ex*. Conversely, if the goal is to identify promising but risky opportunities, the current ranking (favoring higher *Ex*) may be more appropriate. The final ranking can be adjusted according to the decision context and the preferences of stakeholders.

## 7. Analysis and Discussion

This section provides the rigorous analysis and comprehensive discussion of the proposed NCM-LGDM-FMEA approach from multiple perspectives. To enhance clarity, logical flow, and systematic examination, it is structured into four dedicated subsections. Result analysis (Section 7.1) intuitively displays and thoroughly analyzes the outcomes, demonstrating the rationality and effectiveness of the proposed method. Sensitivity analysis (Section 7.2) examines the robustness and applicability of the results by analyzing how variations in input parameters affect the outcomes. Qualitative comparative analysis (Section 7.3) qualitatively compares the proposed method with existing approaches, highlighting its unique contributions and performance gains. Quantitative comparative analysis (Section 7.4) quantitatively compares and analyzes the calculation results and efficiency of the proposed method with those of six other methods. Advantages and limitations analysis (Section 7.5) summarizes the primary advantages and current limitations of our proposed approach. This systematic division facilitates a structured presentation of distinct analytical aspects, ensuring a clear and comprehensive discussion.

### 7.1. Result Analysis

To further substantiate the rationality and effectiveness of the proposed NCM-LGDM-FMEA method, the case study results were visualized, critically analyzed, and discussed. Focusing on FM_1_ as a representative instance, the data preprocessing and weight calculation procedures, along with their derived results, are thoroughly examined.

**Figure 3:**
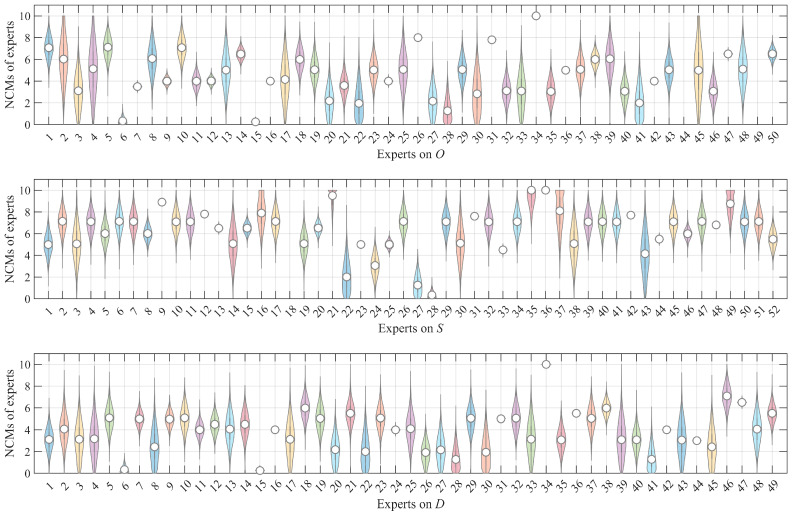
The violin plots of the NCMs of experts’ evaluation data on O, S, and D for FM_1_.

To intuitively characterize the magnitude of uncertainty, the NCM results were illustrated using violin plots. For FM_1_, the violin plots of experts’ heterogeneous evaluation data transformed into NCMs are shown in Figure 3. It can be observed that a larger range in the violin plot corresponds to greater uncertainty. For instance, evaluations by experts 2, 4, and 45 on O, experts 14 and 30 on S, and experts 17 and 33 on D—all of whom employed linguistic expressions with broad coverage to represent their assessments—exhibit higher uncertainties. Conversely, evaluations expressed with an exact number, a narrower interval number, or a single linguistic term tends to demonstrate smaller associated uncertainties. The magnitude of uncertainty directly influences subsequent calculations of data weights. Additionally, as evident in Figure 3, some experts’ NCM violin plots are absent, such as experts 44 and 49 for O and expert 18 for S. This is because these experts provided invalid data, falling outside the predefined universe of discourse, which precluded their transformation into NCMs. Consequently, these invalid data were excluded from all subsequent calculations.

**Figure 4:**
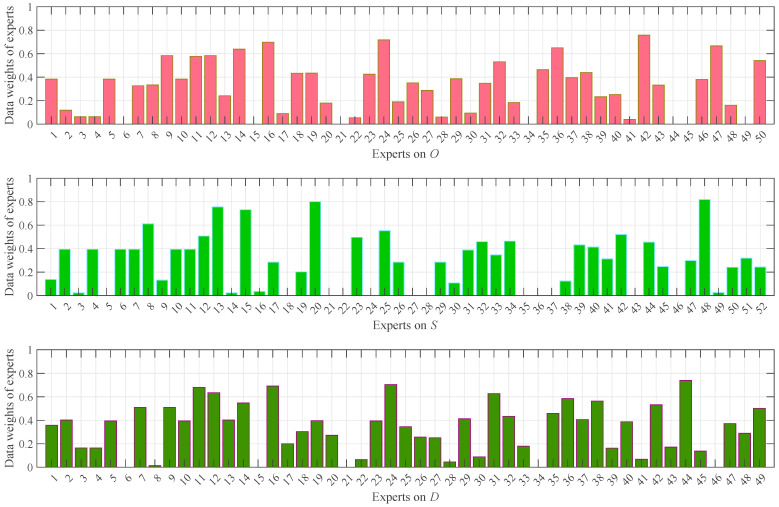
The data weights of experts on O, S, and D for FM_1_.

After removing unreasonable data from the NCM representations of the valid data obtained earlier, the weights of the remaining data were calculated, as shown in Figure 4. It is evident that the smaller the uncertainty of a data item and the closer its expectation to those of other data, the larger its weight. For example, experts 16, 24, and 42 on O, experts 13, 20, and 48 on S, and experts 16, 24, and 44 on D—all of whom evaluated with very low uncertainty and whose expectations were close to those of other experts—exhibit larger weights. Conversely, data with higher uncertainty or greater deviation from others, such as experts 22 and 41 on O, experts 3, 14, and 49 on S, and experts 8 and 28 on D, were assigned smaller weights. It is also important to note the absence of data weights for certain experts, such as experts 6, 15, 21, 34, and 45 on O, experts 5, 21, 22, 27, 28, 35, 36, and 37 on S, and experts 6, 15, 21, 34, and 46 on D. This is due to their data being identified as unreasonable and subsequently removed based on (16), (17), and (18), precluding the calculation of their weights.

**Figure 5:**
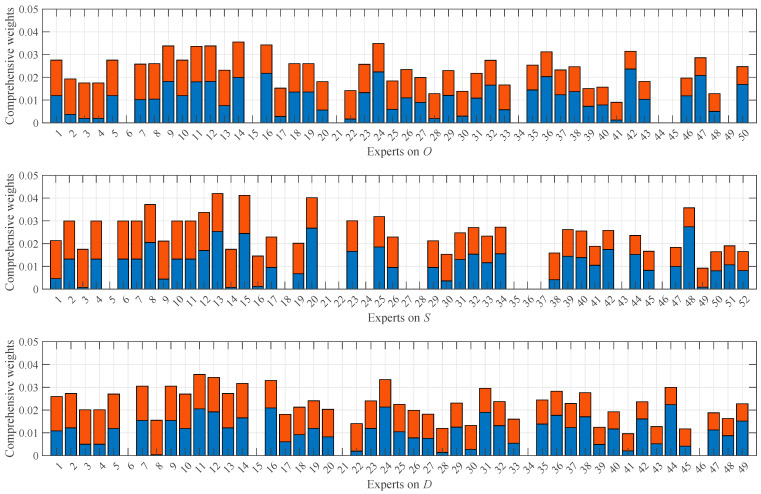
The comprehensive weights combined data weights (light blue below) and identity weights (light red above) of experts on O, S, and D for FM_1_.

The comprehensive weights, which combine data weights and identity weights, were calculated as depicted in Figure 5. With *α* = 0.5, identity weights and data weights generally exhibit comparable magnitudes. This balance enables the comprehensive weights to more effectively reconcile the influence of expert identity and data quality, leading to a more robust and reasonable weight assignment.

The outcomes of the result analysis unambiguously confirm that the data preprocessing and comprehensive weighting procedures functioned as intended. This analysis underscores both the necessity of integrating data preprocessing into LGDM-based FMEA and the advantage of integrating data quality with expert identity to derive more judicious expert weights. Collectively, these findings substantiate the rationale and efficacy of the proposed NCM-LGDM-FMEA method.

### 7.2. Sensitivity Analysis

The proposed NCM-LGDM-FMEA method incorporates a trade-off coefficient, *α*, which balances identity weights and data weights. To examine the robustness of the proposed method and investigate the effects of varying *α* on the results, a sensitivity analysis was performed.

The coefficient *α* influences the calculation of comprehensive weights, thereby ultimately affecting the ranking outcomes. In the case study, *α* was set to 0.5. However, since *α* can vary within the range [0, 1], its sensitivity was thoroughly examined by adjusting it from 0 to 1 in increments of 0.2.

**Figure 6:**
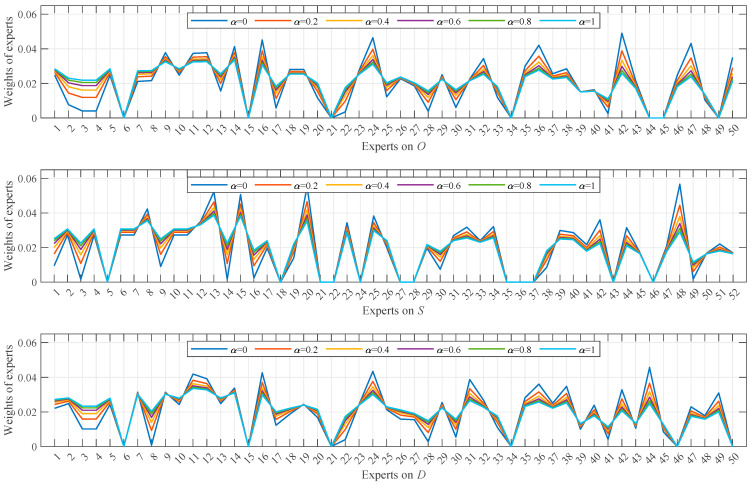
The comprehensive weights of experts under different *α* values.

Figure 6 illustrates the comprehensive weights of experts who evaluated O, S, and D of FM_1_ as *α* varies. The expert weights exhibit minimal variations with changes in *α*, indicating that the weighting method is largely insensitive to this trade-off coefficient, thus demonstrating considerable robustness.

**Figure 7:**
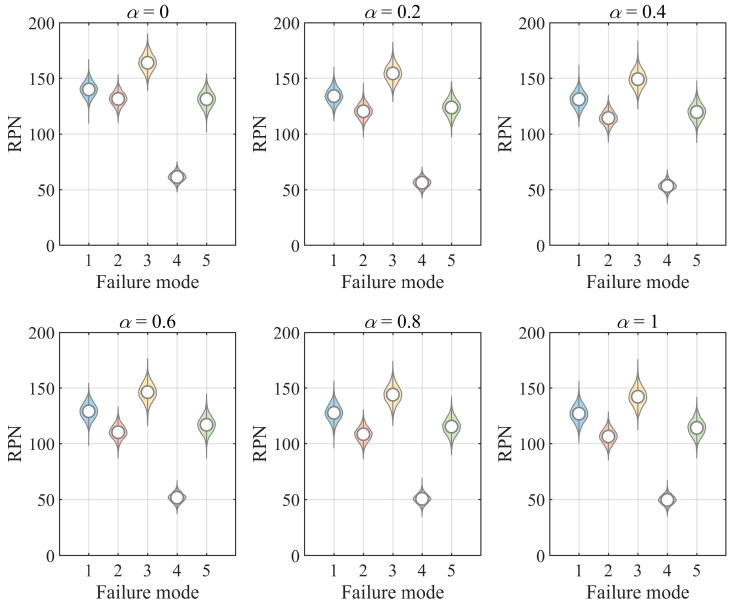
The violin plots of RPN values of the five FMs as *α* varies.

Given that the $\tilde{RPN}$ values and the ranking of FMs are contingent on expert weights, variations in *α* can alter the resulting ranking outcomes. Table 8 and Figure 7 present the RPN values of the five FMs across different *α* values. It can be seen that the ranking results ${\mathrm{FM}}_{3}≻{\mathrm{FM}}_{1}≻{\mathrm{FM}}_{5}≻{\mathrm{FM}}_{2}≻{\mathrm{FM}}_{4}$ remain largely stable, with the only observed variation being the relative order of FM_2_ and FM_5_. Specifically, when *α* = 0, FM_2_ ranks higher than FM_5_; otherwise, FM_5_ ranks higher.

These findings suggest that the outcomes are highly stable with respect to variations in the trade-off coefficient *α*. Thus, the proposed method for calculating expert weights demonstrates substantial robustness. Concerning the final rankings, the sensitivity of the trade-off coefficient *α* is also notably low, leading to stable outcomes. This finding indicates that the proposed NCM-LGDM-FMEA method demonstrates excellent robustness and reliability. Thus, the FMEA framework based on LGDM and NCM proposed in this study exhibits exceptional robustness and applicability.

### 7.3. Qualitative Comparative Analysis

Numerous improved models, such as those based on fuzzy sets, HFS, Z-numbers, and NCM, have been proposed to address FMEA problems under uncertain environments. To the best of the authors’ knowledge, prior studies rarely simultaneously considered multiple types of heterogeneous data, LGDM, and explicit data preprocessing.

This paper, grounded in the LGDM theory, utilizes the NCM to model exact numbers, interval numbers, linguistic terms, and linguistic expressions, implements a four-step data preprocessing process, and proposes a novel expert weighting method. A direct quantitative comparison with existing methods would be unrealistic and unfair, as previous methods generally lack the capacity to handle diverse heterogeneous data types, facilitate LGDM, and integrate data preprocessing, among other limitations. Therefore, to demonstrate the superiority and unique contributions of the proposed NCM-LGDM-FMEA method, a qualitative comparative analysis against a large number of recent methods is conducted, as detailed in Table 9.

Table 9 reveals several key differentiators of our proposed NCM-LGDM-FMEA method.

(1) Data Input and Transformation: Previous studies predominantly relied on linguistic terms or fuzzy set extensions to represent evaluation data. In contrast, our approach accommodates a broader spectrum of heterogeneous data types, including exact numbers, interval numbers, NCMs, linguistic terms, and linguistic expressions. This enhances the flexibility and richness for expressing subjective qualitative opinions. By unifying these diverse data into NCMs, the method effectively manages various uncertainties (e.g., fuzziness, randomness, hesitation) inherent in the decision-making process. Consequently, our approach offers greater inclusiveness and more appropriate model support for input data representation and transformation.

(2) Expert Group Size and Data Preprocessing: Prior research, even in the LGDM contexts, typically involved fewer than 22 experts. However, increasingly complex systems, such as those in aerospace, necessitate FMEA studies that integrate the collective intelligence of large expert groups spanning diverse departments and specializations. This study incorporates over 50 experts, significantly exceeding the scale of previous research. Furthermore, this is notably the first FMEA-related study to integrate comprehensive data preprocessing, which substantially reduces the impact of low-quality data and enhances the scientific rigor of LGDM-based FMEA.

(3) Expert Weight Determination: Only a few previous papers simultaneously considered both subjective and objective aspects for expert weighting. This paper proposes a novel comprehensive weight calculation method that integrates both subjective and objective weights with a single trade-off coefficient. Particularly, the objective weights are quantitatively determined by data quality, utilizing a two-dimensional Gaussian function based on the UD and DD of the data provided by experts. This makes the proposed expert weight calculation method comprehensive, objective, and more rational.

(4) Uncertainty Propagation in Results: Many prior studies, despite dealing with uncertain input data, often failed to retain or propagate this inherent uncertainty during calculations. Their final computational results, used for ranking, were typically converted into exact numbers, losing valuable uncertainty information. In contrast, our method explicitly uses NCM to model uncertain data and maintains uncertainty propagation throughout the entire computational process. As a result, when performing comparison and ranking, not only can clear ranking results be obtained, but the uncertainty levels can also be quantitatively analyzed. Therefore, the proposed NCM-LGDM-FMEA method yields more insightful and information-rich results.

### 7.4. Quantitative Comparative Analysis

To demonstrate the rationality and advantages of the proposed NCM-LGDM-FMEA method for the ADS of modern airplanes, other six FMEA methods were conducted using the same case to obtain the ranking of FMs. Thus, the traditional FMEA [2], NCM-FMEA-2019 [10], NCM-FMEA-2024 [29], NCM-TOPSIS-FMEA-2019 [19], RFIC-TOPSIS-FMEA-2023 [15], NCM-VIKOR-FMEA-2021 [22], and our proposed NCM-LGDM-FMEA all utilized the data from the case study in this paper for quantitative calculation and comparative analysis.

As other methods generally lack the capacity to handle diverse heterogeneous data types, a direct quantitative comparison would be unrealistic and unfair. To compare the differences in methods, it is necessary to ensure the consistency of inputs. Therefore, it is assumed that the original inputs for all methods are derived from the NCMs obtained after preprocessing the heterogeneous data through steps 1 to 4 in this paper. In addition, to eliminate the influence of RF weights on the results, in methods requiring weights for O, S, and D, it is assumed that their weights are equal.

The final quantitative calculation results produced by different methods are summarized in Table 10. It can be seen that all methods consider FM_3_ has the highest risk. The ranking results obtained by the NCM-TOPSIS-FMEA-2019 [19], RFIC-TOPSIS-FMEA-2023 [15], and our proposed NCM-LGDM-FMEA are identical. This indicates that the method proposed in this paper can achieve the same results as complex multi-attribute ranking methods. Furthermore, the proposed NCM-LGDM-FMEA yields $\tilde{RPN}$ that incorporate uncertain information, enabling the acquisition of more comprehensive results. However, there are still existing differences among these methods. None of the traditional FMEA, NCM-FMEA-2019 [19], and NCM-FMEA-2024 [29] adopted complex multi-attribute ranking methods, yet there are subtle differences in their results. It is noteworthy that the ranking results obtained by NCM-VIKOR-FMEA-2021 [22] differ significantly from other methods. This is primarily due to the substantial differences in the ranking method of VIKOR.

**Figure 8:**
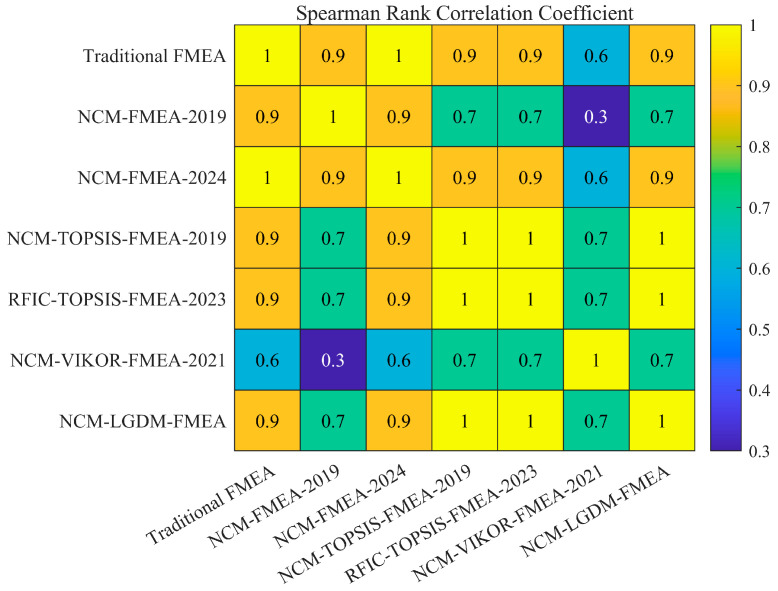
The Spearman rank correlation coefficient matrix of rankings from pairwise comparative methods.

**Figure 9:**
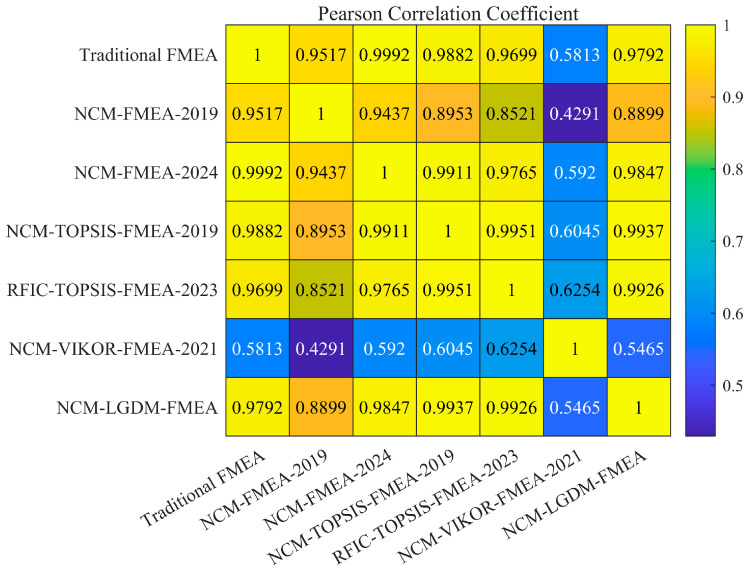
The Pearson correlation coefficient matrix of RPNs from pairwise comparative methods.

To analyze correlations among pairwise comparative methods, the Spearman rank correlation coefficient matrix of rankings and the Pearson correlation coefficient matrix of RPNs (*cd* or *Q*) were calculated, as shown in Figure 8 and Figure 9, respectively. It can be seen that, except for NCM-VIKOR-FMEA-2021 [22], all other methods exhibit a high correlation. This further validates the rationality of the method proposed in this paper.

**Figure 10:**
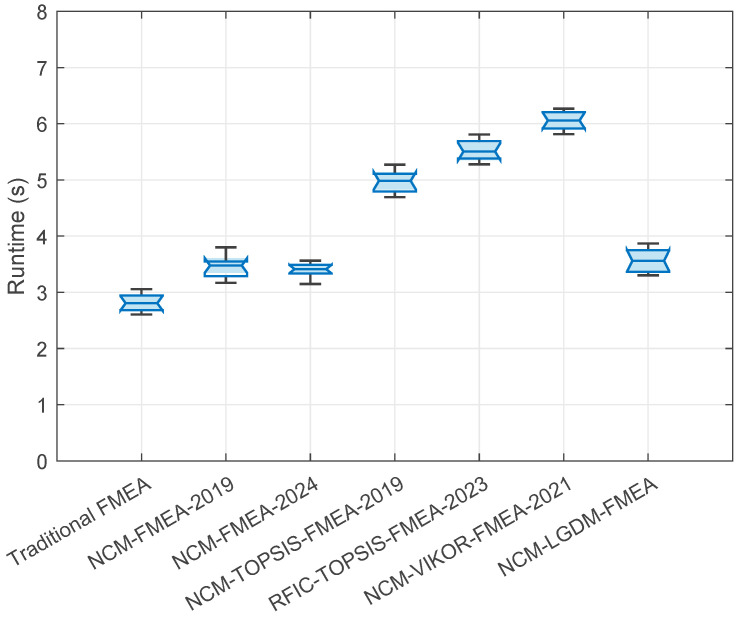
The box plots of runtimes for the seven methods.

To quantitatively compare the time complexity of different methods, each method was repeated 10 times, and the box plots of the runtimes for the seven methods are shown in Figure 10. It can be seen that the time complexity of the proposed NCM-LGDM-FMEA is basically comparable to those classic FMEA method. However, the FMEA methods based on complex multi-attribute ranking, namely NCM-TOPSIS-FMEA-2019 [19], RFIC-TOPSIS-FMEA-2023 [15], and NCM-VIKOR-FMEA-2021 [22], require significantly more computation time. This indicates that the method proposed in this paper can achieve the same results as complex methods while utilizing less time. It is worth noting that the computation time for all methods is within seconds. Therefore, the computation time fully meets the requirements of practical applications.

The quantitative comparison analysis shows that the proposed NCM-LGDM-FMEA is capable of obtaining more comprehensive and rational results in comparison with the traditional FMEA and other recently improved FMEA methods. Compared to complex multi-attribute ranking-based FMEA methods, the proposed NCM-LGDM-FMEA requires less computation time.

### 7.5. Advantages and Limitations Analysis

The practical case study and subsequent analyses strongly validate that our proposed NCM-LGDM-FMEA method, leveraging LGDM and the NCM, establishes a more scientific and robust evaluation framework. The primary advantages are summarized as follows.

(1) Genuine LGDM with Enhanced Flexibility: This method systematically incorporates LGDM into FMEA by facilitating the participation of a large and diverse group of experts. Unlike conventional approaches that require each expert to evaluate every FM and RF, our framework allows experts to focus on specific domains of competence. This design significantly enhances the flexibility and practicality of expert evaluations. In our case study, over 50 experts were engaged—considerably exceeding the scale of typical LGDM-based FMEA applications. Moreover, this research uniquely integrates data preprocessing into LGDM for FMEA, ensuring data cleanliness by effectively filtering invalid and low-quality submissions.

(2) Expanded Data Modeling with Uncertainty Preservation: The NCM is effectively utilized to model a wider and more extensive range of data types. During the evaluation process, experts can flexibly employ exact numbers, interval numbers, NCMs, linguistic terms, and linguistic expressions to represent their assessments across different FMs and RFs. Crucially, different experts can submit disparate data types, and even a single expert can use varying data types across different evaluations. All these diverse inputs are uniformly transformed into NCMs, thereby preserving and propagating uncertainty information throughout the entire computational process. Consequently, this approach not only yields definitive ranking outcomes but also enables comprehensive quantitative uncertainty analysis.

(3) Innovative and Robust Expert Weight Determination: An innovative and comprehensive method for expert weight determination is proposed. This approach incorporates subjective dimensions, such as experts’ work experience and professional titles, to define their relative importance. Simultaneously, an objective calculation of the significance of an expert’s data is achieved using a two-dimensional Gaussian function based on the UD and DD of their provided evaluation data, making it entirely determined by data quality. Importantly, an expert’s weight can vary across FMs and RFs based on the relevance and quality of their specific input. Both the result analysis and sensitivity tests confirm the rationality and robustness of this weighting strategy.

(4) Effective Uncertainty Representation Model: The NCM provides a unified framework to capture fuzziness and randomness of expert judgment, overcoming the limitation of conventional fuzzy sets that assume crisp membership degrees. Using three parameters, NCMs efficiently encapsulate diverse uncertainties. Fuzzy statistics and membership function fitting are applied to model linguistic terms within the NCM framework. In addition, the NCM synthetic operator and the NCM weighted average operator are employed to deal with linguistic expressions and to aggregate multi-expert evaluations, respectively. Thus, the NCM and its aggregation operators provide an effective tool for dealing with the uncertainty, ensuring that uncertainty is preserved and propagated across all computational stages for more informative results.

Alongside these strengths, the proposed method has certain limitations, which also indicate directions for future research.

(1) Refinement of RFs and Their Weights: The current implementation considers only the three conventional RFs: O, S, and D. To overcome this limitation, the framework is inherently extensible. The core methodology is designed to be factor-agnostic. Future work can seamlessly integrate additional RFs by simply expanding the evaluation matrix. Furthermore, it does not take into account the weights for these RFs. To improve discrimination and realism, future work should investigate the integration of specific weighting techniques, for instance, (a) subjective weighting methods (e.g., AHP) can be used to incorporate expert judgment on the relative importance of RFs in different industrial contexts, and (b) objective weighting methods (e.g., CRITIC, entropy-based approaches) could be applied to derive weights directly from historical failure data, making the assessment more data-driven. A hybrid weighting model that combines both subjective and objective approaches would represent a substantial theoretical and practical advancement, offering greater flexibility in environments where both expert judgment and quantitative evidence are available.

(2) Enrichment of Uncertainty Representation Mechanism: Although NCMs provide a robust uncertainty representation, further theoretical extensions could focus on integrating more sophisticated linguistic constructs to capture richer expert knowledge. Two concrete directions are: (a) by integration with probabilistic linguistic term sets, the method could model not only the vagueness of risk assessments but also the distribution or confidence levels among multiple linguistic terms used by experts; (b) by extension to interval rough integrated NCMs, the method can handle situations where risk parameters are given not as precise NCMs but as intervals with rough boundaries, enhancing its applicability to problems with imprecise or incomplete data sources (e.g., early-stage design risk analysis). These extensions would directly address practical needs in complex evaluation contexts where uncertainty is multi-faceted.

## 8. Conclusions

This paper proposes a novel FMEA method that integrates LGDM, heterogeneous data, NCMs, and data preprocessing to evaluate and rank FMs. We significantly enhanced the classical FMEA process by incorporating genuine LGDM, involving a greater number of experts. To accommodate varying expert preferences and the distinct characteristics of FMs and RFs, our method utilizes a broad spectrum of heterogeneous data inputs, including exact numbers, interval numbers, NCMs, linguistic terms, and linguistic expressions. These diverse data are then preprocessed and uniformly converted into NCMs, thereby preserving and propagating their intrinsic uncertainty throughout the analysis. Furthermore, we proposed an innovative and comprehensive expert weight determination method that judiciously combines both subjective and objective factors. The resulting RPN values, represented as NCMs, reflect both magnitude and uncertainty, enabling not only clear ranking but also a comprehensive, quantitative uncertainty analysis.

A practical case study effectively demonstrated the effectiveness, reasonableness, and feasibility of the proposed NCM-LGDM-FMEA method. Result analysis, sensitivity analysis, both qualitative and quantitative comparative analysis, and advantages and limitations analysis collectively confirm the scientific rigor, reasonableness, robustness, and effectiveness of our approach for FMEA, particularly as applied in modern aviation. Our proposed NCM-LGDM-FMEA method excels at processing a wider array of data types and generating more stable and informative results, critically handling and propagating uncertainty information across the entire computational process. This study successfully addresses key challenges in LGDM-based FMEA, specifically the representation and manipulation of diverse uncertainties in heterogeneous data environments, efficient data transformation and preprocessing, and precise determination of expert weights.

This method provides system administrators with a robust, data-driven framework for FMEA. By moving beyond traditional minority decision-making through LGDM integration, it offers a more nuanced understanding of failures and risks. This holistic view empowers administrators to make more informed decisions regarding resource allocation, technological reserves, and risk management, fostering improved strategic alignment between human capital and institutional goals.

Despite its significant advantages, the proposed NCM-LGDM-FMEA method presents limitations that suggest promising directions for future research. While representing a substantial advancement, the current FMEA method did not account for the weights of RFs. Future research should prioritize exploring subjective and objective weighting methods for RFs, such as AHP, CRITIC, and entropy-based approaches. Additionally, incorporating multi-granular and probabilistic linguistic terms into knowledge representation, utilizing NCMs as the underlying modeling framework, holds potential to further enhance the method’s capability in tackling complex real-world problems.

Finally, while this study specifically focuses on FMEA, the proposed method exhibits broader applicability across various practical decision-making problems. Future research will therefore focus on exploring and validating its utility in diverse contexts, including risk assessment, supplier ranking, and project evaluation.

## Figures and Tables

**Table 1:** Nine linguistic terms modeled by NCMs [39,40].

Linguistic Terms	NCMs
*T*_1_: *none* (*n*)	(0.000, 0.100, 0.020)
*T*_2_: *very low* (*vl*)	(0.197, 0.457, 0.022)
*T*_3_: *low*	(1.246, 1.062, 0.034)
*T*_4_: *slightly low* (*sl*)	(3.083, 1.030, 0.033)
*T*_5_: *medium* (*med*)	(5.058, 1.109, 0.056)
*T*_6_: *slightly high* (*sh*)	(7.105, 1.027, 0.041)
*T*_7_: *high*	(8.743, 1.242, 0.041)
*T*_8_: *very high* (*vh*)	(9.775, 1.054, 0.027)
*T*_9_: *maximum* (*m*)	(10.00, 0.100, 0.020)

**Table 2:** Identity classification table.

		Professional Title	
		Senior	Below Senior
Work experience	More than 5 years	G_1_	G_2_
	Less than 5 years	G_3_	G_4_

**Table 3:** Identified FMs of the ADS.

FM	Failure Mode	Failure Effect
FM_1_	ADIRU failure	Lose the function of computing
FM_2_	Pitot tube heating failure	Not available to control the temperature
FM_3_	ADM malfunction	Quantization error or quantization noise
FM_4_	Pitot tube blockage	Lack of speed data
FM_5_	Static port blockage	Inaccurate of altitude data

**Table 4:** The original data provided by experts on FM_1_.

RF	Subgroups and Original Data ^a,b^			
	G_1_	G_2_	G_3_	G_4_
O	*sh* between *sl* and *high* between *low* and *med* between *low* and *high* *sh* *vl* [3, 4] between *med* and *sh* [3, 5] *sh* [2, 6] [3, 5] between *sl* and *sh* [5, 8]	[0, 0.5] 4 between *low* and *sh* (6,1,0.04) *med* between *low* and *sl* (3.6,1,0.2) at most *med* *med* [3.5, 4.5] between *sl* and *sh* 8	between *low* and *sl* lower than *med* *med* at most *sh* 7.8 *sl* between *low* and *med* *m* *sl* 5 *med* [4, 8]	between *med* and *sh* *sl* lower than *sh* 4 *med* 50 between *vl* and *vh* *sl* [6, 7] between *sl* and *sh* better than *sh* [5, 8]
S	[2, 8] *sh* between *sl* and *sh* *sh* (6,1,0.1) *sh* *sh* [4, 8] 8.9 *sh* between *sh* and *sh* 7.8 [6, 7] between *sl* and *sh* [5, 8]	between *sh* and *high* *sh* [50, 60] *med* [5, 8] at least *high* at most *med* 5 *sl* [4, 6] *sh* *low* *vl*	*sh* between *sl* and *sh* 7.6 *sh* [4, 5] between *sh* and *sh* (10,1,0.2) 10 at least *med* between *sl* and *sh* *sh* *sh*	*sh* 7.7 between *low* and *sh* [5, 6] *sh* (6,0.5,0.1) *sh* 6.8 *high* *sh* *sh* [3, 8]
D	*sl* between *sl* and *med* between *low* and *med* between *low* and *med* *med* *vl* [3, 7] between *vl* and *med* [3, 7] *med* [2, 6] [2, 7] between *sl* and *med* [1, 8]	[0, 0.5] 4 between *low* and *med* (6,1,0.04) *med* between *low* and *sl* (5.5,1,0.2) at most *med* *med* [3.5, 4.5] between *sl* and *med* (1.9,1,0.04)	between *low* and *sl* lower than *med* *med* at most *med* 5 *med* between *low* and *med* greater than *vh* *sl* 5.5 *med* [4, 8]	between *low* and *med* *sl* lower than *med* 4 between *low* and *med* 3 between *vl* and *med* *sh* [6, 7] between *sl* and *med* [3, 8]

^a^ The linguistic terms *n*, *vl*, *low*, *sl*, *med*, *sh*, *high*, *vh*, and *m* are defined in Table 1. ^b^ The subgroups G_1_, G_2_, G_3_, and G_4_ are defined in Table 2.

**Table 5:** The NCM representations of original data.

RF	Subgroup			
	G_1_	G_2_	G_3_	G_4_
O	(7.1050,1.0270,0.0410) (5.9973,2.0793,0.0871) (3.1290,1.7208,0.0734) (5.0470,2.4015,0.0935) (7.1050,1.0270,0.0410) (0.1970,0.4570,0.0220) (3.5000,0.1667,0.0000) (6.0815,1.4092,0.0694) (4.0000,0.3333,0.0000) (7.1050,1.0270,0.0410) (4.0000,0.6667,0.0000) (4.0000,0.3333,0.0000) (5.0820,1.6988,0.0769) (6.5000,0.5000,0.0000)	(0.2500,0.0833,0.0000) (4.0000,0.0000,0.0000) (4.1230,2.0210,0.0840) (6.0000,1.0000,0.0400) (5.0580,1.1090,0.0560) (2.1645,1.3522,0.0474) (3.6000,1.0000,0.2000) (1.9168,1.7208,0.0792) (5.0580,1.1090,0.0560) (4.0000,0.1667,0.0000) (5.0820,1.6988,0.0769) (8.0000,0.0000,0.0000)	(2.1645,1.3522,0.0474) (1.1315,1.3522,0.0559) (5.0580,1.1090,0.0560) (2.7815,2.0210,0.0891) (7.8000,0.0000,0.0000) (3.0830,1.0300,0.0330) (3.1290,1.7208,0.0734) (10.0000,0.1000,0.0200) (3.0830,1.0300,0.0330) (5.0000,0.0000,0.0000) (5.0580,1.1090,0.0560) (6.0000,0.6667,0.0000)	(6.0815,1.4092,0.0694) (3.0830,1.0300,0.0330) (1.9168,1.7208,0.0792) (4.0000,0.0000,0.0000) (5.0580,1.1090,0.0560) / ^a^ (5.0296,2.4795,0.0998) (3.0830,1.0300,0.0330) (6.5000,0.1667,0.0000) (5.0820,1.6988,0.0769) / (6.5000,0.5000,0.0000)
S	(5.0000,1.0000,0.0000) (7.1050,1.0270,0.0410) (5.0820,1.6988,0.0769) (7.1050,1.0270,0.0410) (6.0000,1.0000,0.1000) (7.1050,1.0270,0.0410) (7.1050,1.0270,0.0410) (6.0000,0.6667,0.0000) (8.9000,0.0000,0.0000) (7.1050,1.0270,0.0410) (7.1050,1.0270,0.0410) (7.8000,0.0000,0.0000) (6.5000,0.1667,0.0000) (5.0820,1.6988,0.0769) (6.5000,0.5000,0.0000)	(7.9240,1.4075,0.0580) (7.1050,1.0270,0.0410) / (5.0580,1.1090,0.0560) (6.5000,0.5000,0.0000) (9.5060,1.3200,0.0530) (1.9168,1.7208,0.0792) (5.0000,0.0000,0.0000) (3.0830,1.0300,0.0330) (5.0000,0.3333,0.0000) (7.1050,1.0270,0.0410) (1.2460,1.0620,0.0340) (0.1970,0.4570,0.0220)	(7.1050,1.0270,0.0410) (5.0820,1.6988,0.0769) (7.6000,0.0000,0.0000) (7.1050,1.0270,0.0410) (4.5000,0.1667,0.0000) (7.1050,1.0270,0.0410) (10.0000,1.0000,0.2000) (10.0000,0.0000,0.0000) (8.1362,1.8677,0.0873) (5.0820,1.6988,0.0769) (7.1050,1.0270,0.0410) (7.1050,1.0270,0.0410)	(7.1050,1.0270,0.0410) (7.7000,0.0000,0.0000) (4.1230,2.0210,0.0840) (5.5000,0.1667,0.0000) (7.1050,1.0270,0.0410) (6.0000,0.5000,0.1000) (7.1050,1.0270,0.0410) (6.8000,0.0000,0.0000) (8.7430,1.2420,0.0410) (7.1050,1.0270,0.0410) (7.1050,1.0270,0.0410) (5.5000,0.8333,0.0000)
D	(3.0830,1.0300,0.0330) (4.0705,1.3987,0.0650) (3.1290,1.7208,0.0734) (3.1290,1.7208,0.0734) (5.0580,1.1090,0.0560) (0.1970,0.4570,0.0220) (5.0000,0.6667,0.0000) (2.3960,1.7208,0.0766) (5.0000,0.6667,0.0000) (5.0580,1.1090,0.0560) (4.0000,0.6667,0.0000) (4.5000,0.8333,0.0000) (4.0705,1.3987,0.0650) (4.5000,1.1667,0.0000)	(0.2500,0.0833,0.0000) (4.0000,0.0000,0.0000) (3.1290,1.7208,0.0734) (6.0000,1.0000,0.0400) (5.0580,1.1090,0.0560) (2.1645,1.3522,0.0474) (5.5000,1.0000,0.2000) (1.9168,1.7208,0.0792) (5.0580,1.1090,0.0560) (4.0000,0.1667,0.0000) (4.0705,1.3987,0.0650) (1.9000,1.0000,0.0400)	(2.1645,1.3522,0.0474) (1.1315,1.3522,0.0559) (5.0580,1.1090,0.0560) (1.9168,1.7208,0.0792) (5.0000,0.0000,0.0000) (5.0580,1.1090,0.0560) (3.1290,1.7208,0.0734) (10.0000,0.1000,0.0200) (3.0830,1.0300,0.0330) (5.5000,0.0000,0.0000) (5.0580,1.1090,0.0560) (6.0000,0.6667,0.0000)	(3.1290,1.7208,0.0734) (3.0830,1.0300,0.0330) (1.1315,1.3522,0.0559) (4.0000,0.0000,0.0000) (3.1290,1.7208,0.0734) (3.0000,0.0000,0.0000) (2.3960,1.7208,0.0766) (7.1050,1.0270,0.0410) (6.5000,0.1667,0.0000) (4.0705,1.3987,0.0650) (5.5000,0.8333,0.0000)

^a^ The forward slash (/) indicates invalid data at this location, and therefore it was not converted into an NCM.

**Table 6:** The data weights and comprehensive weights.

RF	Subgroup							
	G_1_		G_2_		G_3_		G_4_	
	Data Weights	Comprehensive Weights	Data Weights	Comprehensive Weights	Data Weights	Comprehensive Weights	Data Weights	Comprehensive Weights
O	0.3833 0.1187 0.0627 0.0629 0.3833 / 0.3269 0.3341 0.5835 0.3833 0.5774 0.5835 0.2412 0.6384	0.0276 0.0193 0.0176 0.0176 0.0276 / 0.0258 0.0260 0.0338 0.0276 0.0336 0.0338 0.0231 0.0355	/ ^a^ 0.6982 0.0891 0.4338 0.4344 0.1798 / 0.0541 0.4247 0.7176 0.1900 0.3517	/ 0.0343 0.0153 0.0260 0.0260 0.0181 / 0.0142 0.0257 0.0349 0.0184 0.0234	0.2876 0.0607 0.3868 0.0942 0.3483 0.5309 0.1844 / 0.4637 0.6501 0.3957 0.4399	0.0199 0.0128 0.0230 0.0139 0.0218 0.0275 0.0167 / 0.0254 0.0312 0.0233 0.0246	0.2333 0.2527 0.0394 0.7584 0.3321 / / 0.3809 0.6666 0.1610 / 0.5412	0.0151 0.0157 0.0090 0.0315 0.0182 / / 0.0197 0.0286 0.0128 / 0.0247
S	0.1374 0.3944 0.0236 0.3944 / 0.3944 0.3944 0.6115 0.1315 0.3944 0.3944 0.5070 0.7559 0.0236 0.7315	0.0213 0.0299 0.0175 0.0299 / 0.0299 0.0299 0.0372 0.0211 0.0299 0.0299 0.0337 0.0420 0.0175 0.0412	0.0350 0.2851 / 0.2026 0.8005 / / 0.4967 / 0.5527 0.2851 / /	0.0145 0.0229 / 0.0202 0.0402 / / 0.0300 / 0.0319 0.0229 / /	0.2849 0.1081 0.3891 0.4595 0.3468 0.4641 / / / 0.1238 0.4322 0.4139	0.0212 0.0153 0.0247 0.0271 0.0233 0.0272 / / / 0.0158 0.0262 0.0255	0.3130 0.5208 / 0.4554 0.2468 / 0.2983 0.8183 0.0255 0.2409 0.3190 0.2435	0.0188 0.0258 / 0.0236 0.0166 / 0.0183 0.0357 0.0092 0.0164 0.0190 0.0165
D	0.3581 0.4022 0.1645 0.1645 0.3939 / 0.5087 0.0131 0.5087 0.3939 0.6792 0.6349 0.4022 0.5477	0.0260 0.0273 0.0201 0.0201 0.0270 / 0.0305 0.0155 0.0305 0.0270 0.0357 0.0343 0.0273 0.0317	/ 0.6914 0.1998 0.3038 0.3964 0.2722 / 0.0647 0.3941 0.7043 0.3451 0.2569	/ 0.0330 0.0181 0.0213 0.0241 0.0203 / 0.0141 0.0240 0.0334 0.0225 0.0199	0.2512 0.0453 0.4126 0.0885 0.6275 0.4338 0.1792 / 0.4575 0.5846 0.4061 0.5640	0.0182 0.0120 0.0231 0.0133 0.0296 0.0237 0.0160 / 0.0244 0.0283 0.0229 0.0276	0.1624 0.3869 0.0682 0.5319 0.1710 0.7399 0.1371 / 0.3719 0.2890 0.5021	0.0125 0.0193 0.0096 0.0237 0.0127 0.0299 0.0117 / 0.0188 0.0163 0.0227

^a^ The forward slash (/) signifies removed data at this position, so its weight was not calculated.

**Table 7:** The RPN values and ranking of five FMs.

FM	$\tilde{RPN}$	Ranking
FM_1_	(130.0587,7.1313,0.2900)	2
FM_2_	(112.4176,6.1134,0.2602)	4
FM_3_	(147.6224,7.8024,0.3126)	1
FM_4_	(52.4222,4.0819,0.1767)	5
FM_5_	(118.3090,7.2450,0.2954)	3

**Table 8:** The RPN values of five FMs as α varies.

*α*	FM_1_	FM_2_	FM_3_	FM_4_	FM_5_
0	(140.0138,6.5433,0.2535)	(131.6303,6.1514,0.2676)	(164.1291,7.5337,0.2859)	(61.4781,4.0633,0.1796)	(131.1254,7.0553,0.2755)
0.2	(134.2199,6.7857,0.2708)	(120.3813,6.0650,0.2600)	(154.5118,7.5827,0.2974)	(56.1237,4.0251,0.1753)	(123.6981,7.0849,0.2841)
0.4	(131.1229,7.0313,0.2846)	(114.4443,6.0937,0.2598)	(149.3827,7.7333,0.3082)	(53.3574,4.0621,0.1761)	(119.6914,7.1939,0.2922)
0.6	(129.1954,7.2176,0.2945)	(110.7789,6.1327,0.2607)	(146.1953,7.8645,0.3163)	(51.6694,4.1002,0.1774)	(117.1855,7.2911,0.2982)
0.8	(127.8803,7.3575,0.3017)	(108.2915,6.1672,0.2618)	(144.0228,7.9685,0.3223)	(50.5323,4.1316,0.1786)	(115.4704,7.3692,0.3028)
1	(126.9257,7.4649,0.3072)	(106.4932,6.1957,0.2628)	(142.4472,8.0509,0.3269)	(49.7145,4.1567,0.1796)	(114.2228,7.4315,0.3063)

**Table 9:** Qualitative comparative analysis with recent methods.

Method	Inputs	Data Transformation	Expert Size	Data Preprocessing	Expert Weighting	Evaluation Result	Rank Basis
Huang et al. [20] (2021)	Linguistic terms	IVIFC	8	None	Subjective: experience, technical title, work relevance; Objective: deviation between the true values and experts’ evaluation results.	IVIFC	Exact number
Yu et al. [21] (2021)	Exact numbers, interval numbers, uncertain linguistic terms	Similarity analysis	20	None	Identical	Exact number	Exact number
Yu et al. [22] (2021)	Linguistic terms	NCM	5	None	Subjective: professional position, service time, education level, age; Objective: agreement degree, confidence level.	NCM	Exact number
Subramanian et al. [23] (2022)	Linguistic terms	TrFN	4	None	Not mentioned	Exact number	Exact number
Chen et al. [24] (2023)	RBULI	None	20	None	Number of experts and compactness internally	Exact number	Exact number
Sarwar et al. [15] (2023)	Linguistic terms	TFN	4	None	Identical	NCM	Exact number
Li et al. [25] (2023)	Linguistic terms	Interval NCM	6	None	Identical	Rough NCM	Rough NCM
Zhang et al. [26] (2024)	HFS	NWHFS	13	None	Identical	NWHFS	Exact number
Wan et al. [27] (2024)	PFDHHLTS	PFDHHLTE	22	None	Identical	Exact number	Exact number
Mandal et al. [28] (2024)	LZN	None	6	None	Objective: consensus degree.	Exact number	Exact number
Zhang et al. [29] (2024)	Exact numbers, interval numbers, linguistic terms, linguistic expressions	NCM	4	None	Identical	NCM	NCM
Proposed method	Exact numbers, interval numbers, NCMs, linguistic terms, linguistic expressions	NCM	50+	Invalid data filtering, bad data processing, outlier processing, and tolerance limit processing	Subjective: work experience, professional title; Objective: two-dimensional Gaussian function based on UD and DD.	NCM	NCM

**Table 10:** Quantitative calculation results of different methods.

FM	Traditional FMEA		NCM-FMEA-2019		NCM-FMEA-2024		NCM-TOPSIS-FMEA-2019	
	Catelani et al. [2]		Li et al. [10]		Zhang et al. [29]		Liu et al. [19]	
	RPN	Ranking	$\tilde{RPN}$	Ranking	$\tilde{RPN}$	Ranking	*cd*	Ranking
FM_1_	116.8784	2	(109.2232,6.5558,0.3304)	3	(113.9901,105.7027,16.8207)	2	0.7342	2
FM_2_	106.1373	3	(117.2943,7.0983,0.3525)	2	(102.8871,107.1559,15.5580)	3	0.5931	4
FM_3_	130.5532	1	(126.6412,7.6686,0.3784)	1	(128.9849,113.9134,17.6827)	1	0.8041	1
FM_4_	57.8882	5	(69.7994,5.3821,0.3329)	5	(56.8521,76.1125,10.7470)	5	0.2658	5
FM_5_	96.4307	4	(92.6664,6.0512,0.2939)	4	(96.0154,88.9802,14.6860)	4	0.6034	3
FM	RFIC-TOPSIS-FMEA-2023		NCM-VIKOR-FMEA-2021		NCM-LGDM-FMEA			
	Sarwar et al. [15]		Yu et al. [22]		Proposed method in this paper			
	*cd*	Ranking	*Q*	Ranking	$\tilde{RPN}$	Ranking		
FM_1_	0.6283	2	0.8319	2	(130.0587,7.1313,0.2900)	2		
FM_2_	0.5333	4	0.2843	5	(112.4176,6.1134,0.2602)	4		
FM_3_	0.6703	1	1.0000	1	(147.6224,7.8024,0.3126)	1		
FM_4_	0.3683	5	0.5000	3	(52.4222,4.0819,0.1767)	5		
FM_5_	0.5714	3	0.4390	4	(118.3090,7.2450,0.2954)	3		

## Data Availability

The data presented in this study are available on request from the corresponding author and the Supplementary Materials.

## Supplementary Materials

The following supporting information can be downloaded at: https://www.mdpi.com/article/10.3390/e28030360/s1, Table S1: The original data provided by experts on FM_1_; Table S2: The NCM representations of original data on FM_1_; Table S3: The data weights and comprehensive weights on FM_1_; Table S4: The original data provided by experts on FM_2_; Table S5: The NCM representations of original data on FM_2_; Table S6: The data weights and comprehensive weights on FM_2_; Table S7: The original data provided by experts on FM_3_; Table S8: The NCM representations of original data on FM_3_; Table S9: The data weights and comprehensive weights on FM_3_; Table S10: The original data provided by experts on FM_4_; Table S11: The NCM representations of original data on FM_4_; Table S12: The data weights and comprehensive weights on FM_4_; Table S13: The original data provided by experts on FM_5_; Table S14: The NCM representations of original data on FM_5_; Table S15: The data weights and comprehensive weights on FM_5_.

## Abbreviations

The following abbreviations are used in this manuscript:

FMEA	Failure Modes and Effects Analysis
LGDM	Large Group Decision Making
NCM	Normal Cloud Model
FM	Failure Mode
RPN	Risk Priority Number
O	Occurrence
S	Severity
D	Probability of Non-Detection
RF	Risk Factor
RFIC	Rough Fuzzy Integrated Cloud
TOPSIS	Technique for Order of Preference by Similarity to Ideal Solution
VIKOR	VlseKriterijumska Optimizacija I Kompromisno Resenje
DEMATEL	Decision-Making Trial and Evaluation Laboratory
UD	Uncertainty Degree
DD	Difference Degree
IVIFC	Interval-Valued Intuitionistic Fuzzy Cloud
DEA	Data Envelopment Analysis
TrFN	Trapezoidal Fuzzy Number
RBULI	Relative Basic Uncertain Linguistic Information
TFN	Triangular Fuzzy Number
HFS	Hesitant Fuzzy Set
NWHFS	Normal Wiggly Hesitant Fuzzy Set
PFDHHLTS	Probabilistic Free Double Hierarchy Hesitant Linguistic Term Set
LZN	Linguistic Z-Number
Ex	Expectation
En	Entropy
He	Hyper-entropy
HCLTS	Hesitant Cloud Linguistic Term Set
IQR	Interquartile Range
TIC	Theil’s Inequality Coefficient
ADS	Air Data System
ADM	Air Data Module
ADIRU	Air Data and Inertial Reference Unit
ADR	Air Data Reference
IR	Inertial Reference
ADC	Air Data Computer
