# Supplementary material for: Failure Mode and Effect Analysis Using Large-Scale Group Decision Making and Normal Cloud Model
Source: Entropy (Basel). 2026 Mar 22;28(3):360. doi: 10.3390/e28030360 (PMC13026005; doi:10.3390/e28030360)
Supplement: Supplementary file 1 [file entropy-28-00360-s001.zip › entropy-4193141-supplementary.pdf]

Supplementary Materials for "Failure Mode and Effect Analysis Using Large-scale Group Decision Making and Normal Cloud Model"

Lijie Wu, Changchun Liu, Hanwen Song

I. DATA ON FM_1

Table S1. The original data provided by experts on FM_1

RF	Subgroups and original data			
	G_1	G_2	G_3	G_4
O	*sh*	[0,0.5]	between *low* and *sl*	between *med* and *sh*
	between *sl* and *high*	4	lower than *med*	*sl*
	between *low* and *med*	between *low* and *sh*	*med*	lower than *sh*
	between *low* and *high*	(6,1,0.04)	at most *sh*	4
	sh	*med*	7.8	*med*
	vl	between *low* and *sl*	*sl*	50
	[3,4]	(3.6,1,0.2)	between *low* and *med*	between *vl* and *vh*
	between *med* and *sh*	at most *med*	*m*	*sl*
	[3,5]	*med*	*sl*	[6,7]
	sh	[3.5,4.5]	5	between *sl* and *sh*
	[2,6]	between *sl* and *sh*	*med*	better than *sh*
	[3,5]	8	[4,8]	[5,8]
	between *sl* and *sh*			
	[5,8]			
S	[2,8]	between *sh* and *high*	*sh*	*sh*
	sh	*sh*	between *sl* and *sh*	7.7
	between *sl* and *sh*	[50,60]	7.6	between *low* and *sh*
	sh	*med*	*sh*	[5,6]
	(6,1,0.1)	[5,8]	[4,5]	*sh*
	sh	at least *high*	between *sh* and *sh*	(6,0.5,0.1)
	sh	at most *med*	(10,1,0.2)	*sh*
	[4,8]	5	10	6.8
	8.9	*sl*	at least *med*	*high*
	sh	[4,6]	between *sl* and *sh*	*sh*
	between *sh* and *sh*	*sh*	*sh*	*sh*
	7.8	*low*	*sh*	[3,8]
	[6,7]	*vl*		
	between *sl* and *sh*			
	[5,8]			
D	*sl*	[0,0.5]	between *low* and *sl*	between *low* and *med*
	between *sl* and *med*	4	lower than *med*	*sl*
	between *low* and *med*	between *low* and *med*	*med*	lower than *med*
	between *low* and *med*	(6,1,0.04)	at most *med*	4
	med	*med*	5	between *low* and *med*
	vl	between *low* and *sl*	*med*	3
	[3,7]	(5.5,1,0.2)	between *low* and *med*	between *vl* and *med*
	between *vl* and *med*	at most *med*	greater than *vh*	*sh*
	[3,7]	*med*	*sl*	[6,7]
	med	[3.5,4.5]	5.5	between *sl* and *med*
	[2,6]	between *sl* and *med*	*med*	[3,8]
	[2,7]	(1.9,1,0.04)	[4,8]	
	between *sl* and *med*			
	[1,8]			

Table S2. The NCM representations of original data on FM_1

RF	Subgroup			
	G_1	G_2	G_3	G_4
O	(7.1050,1.0270,0.0410)	(0.2500,0.0833,0.0000)	(2.1645,1.3522,0.0474)	(6.0815,1.4092,0.0694)
	(5.9973,2.0793,0.0871)	(4.0000,0.0000,0.0000)	(1.1315,1.3522,0.0559)	(3.0830,1.0300,0.0330)
	(3.1290,1.7208,0.0734)	(4.1230,2.0210,0.0840)	(5.0580,1.1090,0.0560)	(1.9168,1.7208,0.0792)
	(5.0470,2.4015,0.0935)	(6.0000,1.0000,0.0400)	(2.7815,2.0210,0.0891)	(4.0000,0.0000,0.0000)
	(7.1050,1.0270,0.0410)	(5.0580,1.1090,0.0560)	(7.8000,0.0000,0.0000)	(5.0580,1.1090,0.0560)
	(0.1970,0.4570,0.0220)	(2.1645,1.3522,0.0474)	(3.0830,1.0300,0.0330)	/ [a],
	(3.5000,0.1667,0.0000)	(3.6000,1.0000,0.2000)	(3.1290,1.7208,0.0734)	(5.0296,2.4795,0.0998)
	(6.0815,1.4092,0.0694)	(1.9168,1.7208,0.0792)	(10.0000,0.1000,0.0200)	(3.0830,1.0300,0.0330)
	(4.0000,0.3333,0.0000)	(5.0580,1.1090,0.0560)	(3.0830,1.0300,0.0330)	(6.5000,0.1667,0.0000)
	(7.1050,1.0270,0.0410)	(4.0000,0.1667,0.0000)	(5.0000,0.0000,0.0000)	(5.0820,1.6988,0.0769)
	(4.0000,0.6667,0.0000)	(5.0820,1.6988,0.0769)	(5.0580,1.1090,0.0560)	/
	(4.0000,0.3333,0.0000)	(8.0000,0.0000,0.0000)	(6.0000,0.6667,0.0000)	(6.5000,0.5000,0.0000)
	(5.0820,1.6988,0.0769)			
	(6.5000,0.5000,0.0000)			
S	(5.0000,1.0000,0.0000)	(7.9240,1.4075,0.0580)	(7.1050,1.0270,0.0410)	(7.1050,1.0270,0.0410)
	(7.1050,1.0270,0.0410)	(7.1050,1.0270,0.0410)	(5.0820,1.6988,0.0769)	(7.7000,0.0000,0.0000)
	(5.0820,1.6988,0.0769)	/	(7.6000,0.0000,0.0000)	(4.1230,2.0210,0.0840)
	(7.1050,1.0270,0.0410)	(5.0580,1.1090,0.0560)	(7.1050,1.0270,0.0410)	(5.5000,0.1667,0.0000)
	(6.0000,1.0000,0.1000)	(6.5000,0.5000,0.0000)	(4.5000,0.1667,0.0000)	(7.1050,1.0270,0.0410)
	(7.1050,1.0270,0.0410)	(9.5060,1.3200,0.0530)	(7.1050,1.0270,0.0410)	(6.0000,0.5000,0.1000)
	(7.1050,1.0270,0.0410)	(1.9168,1.7208,0.0792)	(10.0000,1.0000,0.2000)	(7.1050,1.0270,0.0410)
	(6.0000,0.6667,0.0000)	(5.0000,0.0000,0.0000)	(10.0000,0.0000,0.0000)	(6.8000,0.0000,0.0000)
	(8.9000,0.0000,0.0000)	(3.0830,1.0300,0.0330)	(8.1362,1.8677,0.0873)	(8.7430,1.2420,0.0410)
	(7.1050,1.0270,0.0410)	(5.0000,0.3333,0.0000)	(5.0820,1.6988,0.0769)	(7.1050,1.0270,0.0410)
	(7.1050,1.0270,0.0410)	(7.1050,1.0270,0.0410)	(7.1050,1.0270,0.0410)	(7.1050,1.0270,0.0410)
	(7.8000,0.0000,0.0000)	(1.2460,1.0620,0.0340)	(7.1050,1.0270,0.0410)	(5.5000,0.8333,0.0000)
	(6.5000,0.1667,0.0000)	(0.1970,0.4570,0.0220)		
	(5.0820,1.6988,0.0769)			
	(6.5000,0.5000,0.0000)			
D	(3.0830,1.0300,0.0330)	(0.2500,0.0833,0.0000)	(2.1645,1.3522,0.0474)	(3.1290,1.7208,0.0734)
	(4.0705,1.3987,0.0650)	(4.0000,0.0000,0.0000)	(1.1315,1.3522,0.0559)	(3.0830,1.0300,0.0330)
	(3.1290,1.7208,0.0734)	(3.1290,1.7208,0.0734)	(5.0580,1.1090,0.0560)	(1.1315,1.3522,0.0559)
	(3.1290,1.7208,0.0734)	(6.0000,1.0000,0.0400)	(1.9168,1.7208,0.0792)	(4.0000,0.0000,0.0000)
	(5.0580,1.1090,0.0560)	(5.0580,1.1090,0.0560)	(5.0000,0.0000,0.0000)	(3.1290,1.7208,0.0734)
	(0.1970,0.4570,0.0220)	(2.1645,1.3522,0.0474)	(5.0580,1.1090,0.0560)	(3.0000,0.0000,0.0000)
	(5.0000,0.6667,0.0000)	(5.5000,1.0000,0.2000)	(3.1290,1.7208,0.0734)	(2.3960,1.7208,0.0766)
	(2.3960,1.7208,0.0766)	(1.9168,1.7208,0.0792)	(10.0000,0.1000,0.0200)	(7.1050,1.0270,0.0410)
	(5.0000,0.6667,0.0000)	(5.0580,1.1090,0.0560)	(3.0830,1.0300,0.0330)	(6.5000,0.1667,0.0000)
	(5.0580,1.1090,0.0560)	(4.0000,0.1667,0.0000)	(5.5000,0.0000,0.0000)	(4.0705,1.3987,0.0650)
	(4.0000,0.6667,0.0000)	(4.0705,1.3987,0.0650)	(5.0580,1.1090,0.0560)	(5.5000,0.8333,0.0000)
	(4.5000,0.8333,0.0000)	(1.9000,1.0000,0.0400)	(6.0000,0.6667,0.0000)	
	(4.0705,1.3987,0.0650)			
	(4.5000,1.1667,0.0000)			

[a] The forward slash (/) indicates an invalid data at this location, and therefore it was not converted into an NCM.

Table S3. The data weights and comprehensive weights on FM_1

RF	Subgroup							
	G_1		G_2		G_3		G_4	
	Data weights	Comprehensive	Data weights	Comprehensive	Data weights	Comprehensive	Data weights	Comprehensive
O	0.3833	0.0276	/ [a]	/	0.2876	0.0199	0.2333	0.0151
	0.1187	0.0193	0.6982	0.0343	0.0607	0.0128	0.2527	0.0157
	0.0627	0.0176	0.0891	0.0153	0.3868	0.0230	0.0394	0.0090
	0.0629	0.0176	0.4338	0.0260	0.0942	0.0139	0.7584	0.0315
	0.3833	0.0276	0.4344	0.0260	0.3483	0.0218	0.3321	0.0182
	/	/	0.1798	0.0181	0.5309	0.0275	/	/
	0.3269	0.0258	/	/	0.1844	0.0167	/	/
	0.3341	0.0260	0.0541	0.0142	/	/	0.3809	0.0197
	0.5835	0.0338	0.4247	0.0257	0.4637	0.0254	0.6666	0.0286
	0.3833	0.0276	0.7176	0.0349	0.6501	0.0312	0.1610	0.0128
	0.5774	0.0336	0.1900	0.0184	0.3957	0.0233	/	/
	0.5835	0.0338	0.3517	0.0234	0.4399	0.0246	0.5412	0.0247
	0.2412	0.0231						
	0.6384	0.0355						
S	0.1374	0.0213	0.0350	0.0145	0.2849	0.0212	0.3130	0.0188
	0.3944	0.0299	0.2851	0.0229	0.1081	0.0153	0.5208	0.0258
	0.0236	0.0175	/	/	0.3891	0.0247	/	/
	0.3944	0.0299	0.2026	0.0202	0.4595	0.0271	0.4554	0.0236
	/	/	0.8005	0.0402	0.3468	0.0233	0.2468	0.0166
	0.3944	0.0299	/	/	0.4641	0.0272	/	/
	0.3944	0.0299	/	/	/	/	0.2983	0.0183
	0.6115	0.0372	0.4967	0.0300	/	/	0.8183	0.0357
	0.1315	0.0211	/	/	/	/	0.0255	0.0092
	0.3944	0.0299	0.5527	0.0319	0.1238	0.0158	0.2409	0.0164
	0.3944	0.0299	0.2851	0.0229	0.4322	0.0262	0.3190	0.0190
	0.5070	0.0337	/	/	0.4139	0.0255	0.2435	0.0165
	0.7559	0.0420	/	/				
	0.0236	0.0175						
	0.7315	0.0412						
D	0.3581	0.0260	/	/	0.2512	0.0182	0.1624	0.0125
	0.4022	0.0273	0.6914	0.0330	0.0453	0.0120	0.3869	0.0193
	0.1645	0.0201	0.1998	0.0181	0.4126	0.0231	0.0682	0.0096
	0.1645	0.0201	0.3038	0.0213	0.0885	0.0133	0.5319	0.0237
	0.3939	0.0270	0.3964	0.0241	0.6275	0.0296	0.1710	0.0127
	/	/	0.2722	0.0203	0.4338	0.0237	0.7399	0.0299
	0.5087	0.0305	/	/	0.1792	0.0160	0.1371	0.0117
	0.0131	0.0155	0.0647	0.0141	/	/	/	/
	0.5087	0.0305	0.3941	0.0240	0.4575	0.0244	0.3719	0.0188
	0.3939	0.0270	0.7043	0.0334	0.5846	0.0283	0.2890	0.0163
	0.6792	0.0357	0.3451	0.0225	0.4061	0.0229	0.5021	0.0227
	0.6349	0.0343	0.2569	0.0199	0.5640	0.0276		
	0.4022	0.0273						
	0.5477	0.0317						

[a] The forward slash (/) signifies removed data at this position, so its weight was not calculated.

LGDM results for O, S, and D were calculated as (4.8184,0.1558,0.0065), (6.6190,0.1356,0.0053), and (4.0780,0.1601,0.0065) respectively. The RPN value of FM_1 was calculated as (130.0587,7.1313,0.2900) by multiplying O, S, and D.

II. DATA ON FM_2

Table S4. The original data provided by experts on FM_2

RF	Subgroups and original data			
	G_1	G_2	G_3	G_4
O	*med*<br>lower than *med*<br>between *low* and *med*<br>*high*<br>*sh*<br>*sh*<br>[4,5]<br>between *vl* and *med*<br>5.5<br>*low*<br>[5,8]	[0,0.5]<br>[5,7]<br>at most *med*<br>(3,1,0.04)<br>*med*<br>*sl*<br>(8,1,0.2)<br>at most *sl*<br>*sl*<br>[3.5,4.5]<br>between *sl* and *sh*<br>[5,7]<br>*med*<br>*sh*	between *low* and *sl*<br>lower than *sl*<br>*med*<br>at most *med*<br>between *low* and *sl*<br>*sl*<br>at most *sh*<br>greater than *med*<br>*med*<br>*sh*<br>*med*<br>[0,2]<br>between *low* and *sh*	greater than *med*<br>*sl*<br>lower than *sh*<br>*sh*<br>*high*<br>65<br>*low*<br>*high*<br>(5.8,0.5,0.05)<br>better than *sh*<br>(6.8,0.5,0.05)<br>lower than *sh*<br>(7.8,0.5,0.05)
S	*high*<br>lower than *sl*<br>between *low* and *med*<br>between *med* and *high*<br>*high*<br>*high*<br>[4,5]<br>between *vl* and *med*<br>8.5<br>*low*<br>[5,6]	[0,0.5]<br>[6-9]<br>at most *med*<br>(5,1,0.04)<br>*med*<br>*med*<br>(8,1,0.2)<br>at most *med*<br>*sl*<br>[3.5,4.5]<br>between *sl* and *sh*<br>[5,7]<br>*med*<br>[1,3]	between *low* and *sl*<br>lower than *high*<br>*med*<br>lower than *high*<br>between *low* and *sl*<br>*sl*<br>greater than *sh*<br>greater than *sh*<br>*vl*<br>8<br>*med*<br>[4,9]<br>between *low* and *sh*	between *low* and *sl*<br>*sl*<br>lower than *sh*<br>10<br>lower than *sl*<br>3<br>between *low* and *sl*<br>*sl*<br>*m*<br>better than *med*<br>(2.8,0.5,0.05)<br>lower than *sl*<br>(3.8,0.5,0.05)
D	*high*<br>between *low* and *med*<br>lower than *high*<br>between *low* and *med*<br>*Sh*<br>*vl*<br>[1,5]<br>between *med* and *sh*<br>2<br>*sl*<br>[2,5]	[5,7]<br>6<br>greater than *sh*<br>(5,1,0.04)<br>*med*<br>greater than *med*<br>(3,1,0.2)<br>at most *med*<br>greater than *sh*<br>[1.5,4.5]<br>between *sl* and *sh*<br>[5,9]<br>*med*<br>*med*	between *low* and *sl*<br>lower than *high*<br>*high*<br>at most *sh*<br>3<br>*sh*<br>between *low* and *med*<br>greater than *sh*<br>*sh*<br>22<br>*med*<br>[4,8]<br>between *low* and *sh*	*m*<br>*high*<br>greater than *vl*<br>5<br>between *low* and *med*<br>1<br>between *vl* and *vh*<br>*high*<br>[6,7]<br>between *low* and *med*<br>[5,8]<br>better than *sh*<br>(5.8,0.5,0.05)

Table S5. The NCM representations of original data on FM_2

RF	Subgroup			
	G_1	G_2	G_3	G_4
O	(5.0580,1.1090,0.0560)	(0.2500,0.0833,0.0000)	(2.1645,1.3522,0.0474)	(8.9058,1.4855,0.0670)
	(0.4810,1.0620,0.0452)	(6.0000,0.3333,0.0000)	(0.4810,1.0620,0.0452)	(3.0830,1.0300,0.0330)
	(3.1290,1.7208,0.0734)	(1.9168,1.7208,0.0792)	(5.0580,1.1090,0.0560)	(1.9168,1.7208,0.0792)
	(8.7430,1.2420,0.0410)	(3.0000,1.0000,0.0400)	(1.9168,1.7208,0.0792)	(7.1050,1.0270,0.0410)
	(7.1050,1.0270,0.0410)	(5.0580,1.1090,0.0560)	(2.1645,1.3522,0.0474)	(8.7430,1.2420,0.0410)
	(7.1050,1.0270,0.0410)	(3.0830,1.0300,0.0330)	(3.0830,1.0300,0.0330)	/ [a]
	(4.5000,0.1667,0.0000)	(8.0000,1.0000,0.2000)	(2.7815,2.0210,0.0891)	(1.2460,1.0620,0.0340)
	(2.3960,1.7208,0.0766)	(1.1315,1.3522,0.0559)	(8.9058,1.4855,0.0670)	(8.7430,1.2420,0.0410)
	(5.5000,0.0000,0.0000)	(3.0830,1.0300,0.0330)	(5.0580,1.1090,0.0560)	(5.8000,0.5000,0.0500)
	(1.2460,1.0620,0.0340)	(4.0000,0.1667,0.0000)	(7.1050,1.0270,0.0410)	/
	(6.5000,0.5000,0.0000)	(5.0820,1.6988,0.0769)	(5.0580,1.1090,0.0560)	(6.8000,0.5000,0.0500)
		(6.0000,0.3333,0.0000)	(1.0000,0.3333,0.0000)	(0.4810,1.0620,0.0452)
		(5.0580,1.1090,0.0560)	(4.1230,2.0210,0.0840)	(7.8000,0.5000,0.0500)
		(7.1050,1.0270,0.0410)		
S	(8.7430,1.2420,0.0410)	(0.2500,0.0833,0.0000)	(2.1645,1.3522,0.0474)	(2.1645,1.3522,0.0474)
	(0.4810,1.0620,0.0452)	/	(2.7815,2.0210,0.0891)	(3.0830,1.0300,0.0330)
	(3.1290,1.7208,0.0734)	(1.9168,1.7208,0.0792)	(5.0580,1.1090,0.0560)	(1.9168,1.7208,0.0792)
	(5.0580,1.1090,0.0560)	(5.0000,1.0000,0.0400)	(2.7815,2.0210,0.0891)	(10.0000,0.0000,0.0000)
	(8.7430,1.2420,0.0410)	(5.0580,1.1090,0.0560)	(2.1645,1.3522,0.0474)	(0.4810,1.0620,0.0452)
	(8.7430,1.2420,0.0410)	(5.0580,1.1090,0.0560)	(3.0830,1.0300,0.0330)	(3.0000,0.0000,0.0000)
	(4.5000,0.1667,0.0000)	(8.0000,1.0000,0.2000)	(9.5060,1.3200,0.0530)	(5.0820,1.6988,0.0769)
	(2.3960,1.7208,0.0766)	(1.9168,1.7208,0.0792)	(9.5060,1.3200,0.0530)	(3.0830,1.0300,0.0330)
	(8.5000,0.0000,0.0000)	(3.0830,1.0300,0.0330)	(0.1970,0.4570,0.0220)	(10.0000,0.1000,0.0200)
	(1.2460,1.0620,0.0340)	(4.0000,0.1667,0.0000)	(8.0000,0.0000,0.0000)	/
	(5.5000,0.1667,0.0000)	(5.0820,1.6988,0.0769)	(5.0580,1.1090,0.0560)	(2.8000,0.5000,0.0500)
		(6.0000,0.3333,0.0000)	(6.5000,0.8333,0.0000)	(0.4810,1.0620,0.0452)
		(5.0580,1.1090,0.0560)	(4.1230,2.0210,0.0840)	(3.8000,0.5000,0.0500)
		(2.0000,0.3333,0.0000)		
D	(8.7430,1.2420,0.0410)	(6.0000,0.3333,0.0000)	(2.1645,1.3522,0.0474)	(10.0000,0.1000,0.0200)
	(3.1290,1.7208,0.0734)	(6.0000,0.0000,0.0000)	(2.7815,2.0210,0.0891)	(8.7430,1.2420,0.0410)
	(2.7815,2.0210,0.0891)	(9.5060,1.3200,0.0530)	(8.7430,1.2420,0.0410)	(6.4300,2.4795,0.0994)
	(3.1290,1.7208,0.0734)	(5.0000,1.0000,0.0400)	(2.7815,2.0210,0.0891)	(5.0000,0.0000,0.0000)
	(7.1050,1.0270,0.0410)	(5.0580,1.1090,0.0560)	(3.0000,0.0000,0.0000)	(3.1290,1.7208,0.0734)
	(0.1970,0.4570,0.0220)	(8.9058,1.4855,0.0670)	(7.1050,1.0270,0.0410)	(1.0000,0.0000,0.0000)
	(3.0000,0.6667,0.0000)	(3.0000,1.0000,0.2000)	(3.1290,1.7208,0.0734)	(5.0296,2.4795,0.0998)
	(6.0815,1.4092,0.0694)	(1.9168,1.7208,0.0792)	(9.5060,1.3200,0.0530)	(8.7430,1.2420,0.0410)
	(2.0000,0.0000,0.0000)	(9.5060,1.3200,0.0530)	(7.1050,1.0270,0.0410)	(6.5000,0.1667,0.0000)
	(3.0830,1.0300,0.0330)	(3.0000,0.5000,0.0000)	/	(3.1290,1.7208,0.0734)
	(3.5000,0.5000,0.0000)	(5.0820,1.6988,0.0769)	(5.0580,1.1090,0.0560)	(6.5000,0.5000,0.0000)
		(7.0000,0.6667,0.0000)	(6.0000,0.6667,0.0000)	/
		(5.0580,1.1090,0.0560)	(4.1230,2.0210,0.0840)	(5.8000,0.5000,0.0300)
		(5.0580,1.1090,0.0560)		

[a] The forward slash (/) indicates an invalid data at this location, and therefore it was not converted into an NCM.

Table S6. The data weights and comprehensive weights on FM_2

RF	Subgroup							
	G_1		G_2		G_3		G_4	
	Data weights	Comprehensive	Data weights	Comprehensive	Data weights	Comprehensive	Data weights	Comprehensive
	0.4071	0.0263	0.0141	0.0120	0.2802	0.0182	0.2190	0.0136
	0.0401	0.0156	0.5814	0.0284	0.1499	0.0145	0.3506	0.0174
	0.1457	0.0187	0.0917	0.0142	0.4242	0.0224	0.1300	0.0110
	0.2795	0.0226	0.4333	0.0241	0.2222	0.0166	0.4617	0.0206
	0.4179	0.0266	0.3774	0.0225	0.4118	0.0221	0.3796	0.0182
	0.4179	0.0266	0.4414	0.0244	0.5472	0.0260	/	/
O	0.7594	0.0365	/ [a]	/	/	/	0.2312	0.0139
	0.1199	0.0180	0.0882	0.0141	0.1626	0.0148	0.3413	0.0171
	0.7609	0.0365	0.4482	0.0246	0.4719	0.0238	0.6880	0.0272
	0.1600	0.0191	0.7206	0.0325	0.3984	0.0217	/	/
	0.6804	0.0342	0.1406	0.0157	0.4941	0.0244	0.7068	0.0277
			0.7051	0.0320	0.4128	0.0221	0.0836	0.0097
			0.3844	0.0227	/	/	0.6918	0.0273
			0.3595	0.0220				
	0.3137	0.0238	0.0162	0.0122	0.3028	0.0191	0.2812	0.0156
	0.0590	0.0164	/	/	0.1901	0.0158	0.4816	0.0214
	0.1462	0.0189	0.1037	0.0148	0.5257	0.0257	0.1636	0.0121
	0.4070	0.0266	0.4356	0.0245	0.1785	0.0155	/	/
	0.3137	0.0238	0.3463	0.0219	0.3397	0.0202	0.1684	0.0123
	0.3137	0.0238	0.3463	0.0219	0.5305	0.0258	0.6352	0.0259
S	0.7467	0.0365	/	/	0.3260	0.0198	0.1380	0.0114
	0.1224	0.0182	0.1042	0.0148	0.3102	0.0193	0.4447	0.0204
	0.6746	0.0344	0.4104	0.0237	0.0285	0.0111	/	/
	0.1813	0.0200	0.7534	0.0338	0.6072	0.0280	/	/
	0.7650	0.0371	0.1438	0.0159	0.5108	0.0252	0.6060	0.0251
			0.7435	0.0335	0.5890	0.0275	0.1645	0.0121
			0.4224	0.0241	0.2030	0.0162	0.5744	0.0242
			0.6145	0.0297				
	0.2398	0.0214	0.7066	0.0320	0.2388	0.0170	0.5387	0.0228
	0.2157	0.0207	0.8026	0.0348	0.1714	0.0151	0.1612	0.0119
	0.1200	0.0179	0.2190	0.0179	0.2721	0.0180	/	/
	0.2157	0.0207	0.5258	0.0268	0.1800	0.0153	0.7474	0.0288
	0.3815	0.0255	0.4243	0.0238	0.5045	0.0247	0.0285	0.0080
	0.0191	0.0150	0.1230	0.0151	0.4408	0.0229	0.0768	0.0094
D	0.6866	0.0343	/	/	0.3073	0.0190	/	/
	0.2818	0.0226	0.0066	0.0118	0.2228	0.0166	0.1564	0.0117
	0.5052	0.0291	0.2164	0.0178	0.4300	0.0225	0.7911	0.0301
	0.5265	0.0297	0.3058	0.0204	/	/	0.0275	0.0080
	0.7435	0.0359	0.2072	0.0176	0.5653	0.0265	0.7006	0.0275
			0.6915	0.0316	0.6566	0.0291	/	/
			0.4657	0.0250	0.2401	0.0171	0.6411	0.0258
			0.4657	0.0250				

[a] The forward slash (/) signifies removed data at this position, so its weight was not calculated.

LGDM results for O, S, and D were calculated as (4.7578,0.1463,0.0064), (4.4793,0.1524,0.0065), and (5.2749,0.1542,0.0064) respectively. The RPN value of FM_2 was calculated as (112.4176,6.1134,0.2602)by multiplying O, S, and D.

III. DATA ON FM_3

Table S7. The original data provided by experts on FM_3

RF	Subgroups and original data			
	G_1	G_2	G_3	G_4
	sl	[0,0.5]	between *low* and *sl*	between *med* and *sh*
	between *sl* and *med*	4	lower than *med*	*sl*
	between *low* and *med*	between *low* and *sh*	*med*	lower than *sh*
	between *low* and *med*	(6,1,0.04)	at most *sh*	4
	sh	*med*	7.7	between *low* and *med*
O	*vl*	between *low* and *sl*	*sh*	8
	[3,7]	(7,1,0.2)	between *low* and *med*	between *vl* and *vh*
	between *med* and *sh*	at most *sh*	greater than *vh*	*sl*
	[3,7]	*sl*	*sl*	[6,7]
	sh	[3.5,4.5]	5	between *sl* and *sh*
	[2,6]	between *sl* and *sh*	*med*	[5,8]
		8	[4,8]	better than *sh*
	[2,8]	between *sh* and *high*	*sh*	*sh*
	sh	*sh*	between *sl* and *sh*	*sh*
	between *sl* and *sh*	[50,60]	*sh*	between *low* and *sh*
	sh	*med*	*sh*	[5,6]
	(6,1,0.1)	[5,8]	[4,5]	*sh*
S	*sh*	at least *sh*	between *sh* and *sh*	(6,0.5,0.1)
	sh	at most *med*	(5,1,0.2)	*sh*
	[4,8]	5	10	*sh*
	5	*sl*		*high*
	sh	[4,6]	between *sl* and *sh*	*sh*
	between *sh* and *sh*	*sh*	sh	*sh*
	7		sh	[3,8]
	sl	[0,0.5]	between *low* and *sl*	between *med* and *sh*
	between *sl* and *sh*	4	lower than *med*	*sl*
	between *low* and *med*	between *low* and *sh*	*med*	lower than *sh*
	between *low* and *med*	(6,1,0.04)	at most *med*	4
	sh	*med*	5	between *low* and *med*
D	*vl*	between *low* and *sl*	*sh*	7.5
	[3,7]	(5.5,1,0.2)	between *low* and *med*	between *vl* and *med*
	between *med* and *sh*	at most *sh*	greater than *vh*	*sl*
	[3,7]	*sh*	*sl*	[6,7]
	sh	[3.5,4.5]	5.5	between *sl* and *sh*
	[2,6]	between *sl* and *sh*	*med*	[5,8]
		(4.9,1,0.04)	[4,8]	better than *sh*

Table S8. The NCM representations of original data on FM_3

RF	Subgroup			
	G_1	G_2	G_3	G_4
O	(3.0830,1.0300,0.0330)	(0.2500,0.0833,0.0000)	(2.1645,1.3522,0.0474)	(6.0815,1.4092,0.0694)
	(4.0705,1.3987,0.0650)	(4.0000,0.0000,0.0000)	(1.1315,1.3522,0.0559)	(3.0830,1.0300,0.0330)
	(3.1290,1.7208,0.0734)	(4.1230,2.0210,0.0840)	(5.0580,1.1090,0.0560)	(1.9168,1.7208,0.0792)
	(3.1290,1.7208,0.0734)	(6.0000,1.0000,0.0400)	(2.7815,2.0210,0.0891)	(4.0000,0.0000,0.0000)
	(7.1050,1.0270,0.0410)	(5.0580,1.1090,0.0560)	(7.7000,0.0000,0.0000)	(3.1290,1.7208,0.0734)
	(0.1970,0.4570,0.0220)	(2.1645,1.3522,0.0474)	(7.1050,1.0270,0.0410)	(8.0000,0.0000,0.0000)
	(5.0000,0.6667,0.0000)	(7.0000,1.0000,0.2000)	(3.1290,1.7208,0.0734)	(5.0296,2.4795,0.0998)
	(6.0815,1.4092,0.0694)	(2.7815,2.0210,0.0891)	(10.0000,0.1000,0.0200)	(3.0830,1.0300,0.0330)
	(5.0000,0.6667,0.0000)	(3.0830,1.0300,0.0330)	(3.0830,1.0300,0.0330)	(6.5000,0.1667,0.0000)
	(7.1050,1.0270,0.0410)	(4.0000,0.1667,0.0000)	(5.0000,0.0000,0.0000)	(5.0820,1.6988,0.0769)
	(4.0000,0.6667,0.0000)	(5.0820,1.6988,0.0769)	(5.0580,1.1090,0.0560)	(6.5000,0.5000,0.0000)
		(8.0000,0.0000,0.0000)	(6.0000,0.6667,0.0000)	/ [a]
S	(5.0000,1.0000,0.0000)	(7.9240,1.4075,0.0580)	(7.1050,1.0270,0.0410)	(7.1050,1.0270,0.0410)
	(7.1050,1.0270,0.0410)	(7.1050,1.0270,0.0410)	(5.0820,1.6988,0.0769)	(7.1050,1.0270,0.0410)
	(5.0820,1.6988,0.0769)	/	(7.1050,1.0270,0.0410)	(4.1230,2.0210,0.0840)
	(7.1050,1.0270,0.0410)	(5.0580,1.1090,0.0560)	(7.1050,1.0270,0.0410)	(5.5000,0.1667,0.0000)
	(6.0000,1.0000,0.1000)	(6.5000,0.5000,0.0000)	(4.5000,0.1667,0.0000)	(7.1050,1.0270,0.0410)
	(7.1050,1.0270,0.0410)	(8.9058,1.4855,0.0670)	(7.1050,1.0270,0.0410)	(6.0000,0.5000,0.1000)
	(7.1050,1.0270,0.0410)	(1.9168,1.7208,0.0792)	(5.0000,1.0000,0.2000)	(7.1050,1.0270,0.0410)
	(6.0000,0.6667,0.0000)	(5.0000,0.0000,0.0000)	(10.0000,0.0000,0.0000)	(7.1050,1.0270,0.0410)
	(5.0000,0.0000,0.0000)	(3.0830,1.0300,0.0330)	/	(8.7430,1.2420,0.0410)
	(7.1050,1.0270,0.0410)	(5.0000,0.3333,0.0000)	(5.0820,1.6988,0.0769)	(7.1050,1.0270,0.0410)
	(7.1050,1.0270,0.0410)	(7.1050,1.0270,0.0410)	(7.1050,1.0270,0.0410)	(7.1050,1.0270,0.0410)
	(7.0000,0.0000,0.0000)		(7.1050,1.0270,0.0410)	(5.5000,0.8333,0.0000)
D	(3.0830,1.0300,0.0330)	(0.2500,0.0/833,0.0000)	(2.1645,1.3522,0.0474)	(6.0815,1.4092,0.0694)
	(5.0820,1.6988,0.0769)	(4.0000,0.0000,0.0000)	(1.1315,1.3522,0.0559)	(3.0830,1.0300,0.0330)
	(3.1290,1.7208,0.0734)	(4.1230,2.0210,0.0840)	(5.0580,1.1090,0.0560)	(1.9168,1.7208,0.0792)
	(3.1290,1.7208,0.0734)	(6.0000,1.0000,0.0400)	(1.9168,1.7208,0.0792)	(4.0000,0.0000,0.0000)
	(7.1050,1.0270,0.0410)	(5.0580,1.1090,0.0560)	(5.0000,0.0000,0.0000)	(3.1290,1.7208,0.0734)
	(0.1970,0.4570,0.0220)	(2.1645,1.3522,0.0474)	(7.1050,1.0270,0.0410)	(7.5000,0.0000,0.0000)
	(5.0000,0.6667,0.0000)	(5.5000,1.0000,0.2000)	(3.1290,1.7208,0.0734)	(2.3960,1.7208,0.0766)
	(6.0815,1.4092,0.0694)	(2.7815,2.0210,0.0891)	(10.0000,0.1000,0.0200)	(3.0830,1.0300,0.0330)
	(5.0000,0.6667,0.0000)	(7.1050,1.0270,0.0410)	(3.0830,1.0300,0.0330)	(6.5000,0.1667,0.0000)
	(7.1050,1.0270,0.0410)	(4.0000,0.1667,0.0000)	(5.5000,0.0000,0.0000)	(5.0820,1.6988,0.0769)
	(4.0000,0.6667,0.0000)	(5.0820,1.6988,0.0769)	(5.0580,1.1090,0.0560)	(6.5000,0.5000,0.0000)
		(4.9000,1.0000,0.0400)	(6.0000,0.6667,0.0000)	/

[a] The forward slash (/) indicates an invalid data at this location, and therefore it was not converted into an NCM.

Table S9. The data weights and comprehensive weights on FM_3

RF	Subgroup							
	G_1		G_2		G_3		G_4	
	Data weights	Comprehensive	Data weights	Comprehensive	Data weights	Comprehensive	Data weights	Comprehensive
O	0.3151	0.0262	0.0056	0.0131	0.1575	0.0163	0.2049	0.0146
	0.3685	0.0279	0.7770	0.0379	0.0484	0.0128	0.2687	0.0167
	0.1417	0.0206	0.0835	0.0156	0.4516	0.0258	0.0461	0.0095
	0.1417	0.0206	0.4287	0.0267	0.0933	0.0143	0.7136	0.0310
	0.2483	0.0241	0.4020	0.0258	0.4758	0.0266	0.1110	0.0116
	/ [a]	/	0.2213	0.0200	0.3866	0.0237	0.4490	0.0225
	0.7025	0.0387	/	/	0.1954	0.0175	/	/
	0.2883	0.0254	0.0707	0.0151	/	/	0.3480	0.0192
	0.7025	0.0387	0.4549	0.0275	0.4884	0.0270	0.6604	0.0293
	0.2483	0.0241	0.7817	0.0380	0.7092	0.0341	0.1453	0.0127
	0.6720	0.0377	0.1604	0.0180	0.4418	0.0255	0.6419	0.0287
			0.5318	0.0300	0.5456	0.0288	/	/
S	0.0709	0.0241	/	/	0.3029	0.0275	0.4890	0.0311
	0.3209	0.0346	0.1481	0.0231	/	/	0.4890	0.0311
	/	/	/	/	0.4918	0.0355	/	/
	0.3209	0.0346	0.1197	0.0219	0.3535	0.0296	0.1430	0.0166
	/	/	0.7270	0.0475	0.0620	0.0174	0.1784	0.0181
	0.3209	0.0346	/	/	0.4262	0.0327	/	/
	0.3209	0.0346	/	/	/	/	0.4890	0.0311
	0.5914	0.0460	0.5878	0.0416	/	/	0.4709	0.0304
	0.0650	0.0238	/	/	/	/	0.0472	0.0125
	0.3209	0.0346	0.4926	0.0376	/	/	0.4890	0.0311
	0.3209	0.0346	0.1481	0.0231	0.3044	0.0276	0.4890	0.0311
	0.6714	0.0494			0.4116	0.0321	0.1979	0.0189
D	0.2872	0.0255	/	/	0.2326	0.0189	0.2240	0.0153
	0.2690	0.0249	0.6374	0.0336	0.0513	0.0130	0.3514	0.0195
	0.1376	0.0206	0.1589	0.0181	0.4228	0.0250	0.0701	0.0104
	0.1376	0.0206	0.4608	0.0279	0.0968	0.0145	0.6161	0.0280
	0.2866	0.0255	0.4429	0.0273	0.6843	0.0335	0.1704	0.0136
	/	/	0.0967	0.0161	0.3362	0.0222	0.4709	0.0233
	0.7140	0.0393	/	/	0.1826	0.0172	0.1077	0.0116
	0.3182	0.0265	0.0744	0.0154	/	/	0.4408	0.0224
	0.7140	0.0393	0.3059	0.0228	0.4436	0.0257	0.5653	0.0264
	0.2866	0.0255	0.5869	0.0319	0.6431	0.0321	0.1888	0.0142
	0.6569	0.0374	0.2672	0.0216	0.4126	0.0247	0.5944	0.0273
			0.5994	0.0323	0.5581	0.0294	/	/

[a] The forward slash (/) signifies removed data at this position, so its weight was not calculated.

LGDM results for O, S, and D were calculated as (4.7243,0.1535,0.0062), (6.6263,0.1566,0.0060), and (4.7157,0.1619,0.0066) respectively. The RPN value of FM_3 was calculated as (147.6224,7.8024,0.3126) by multiplying O, S, and D.

IV. DATA ON FM_4

Table S10. The original data provided by experts on FM_4

RF	Subgroups and original data			
	G_1	G_2	G_3	G_4
	high	[0,0.5]	between *low* and *sl*	between *low* and *sl*
	lower than *sl*	[6-9]	lower than *sl*	*sl*
	between *low* and *med*	at most *med*	*med*	lower than *sh*
	between *sl* and *hign*	(3,1,0.04)	at most *med*	4
	vl	*med*	between *low* and *sl*	lower than *sl*
	vl	*sl*	*sl*	65
O	[1,5]	(8,1,0.2)	at most *sh*	between *sl* and *sh*
	between *vl* and *med*	at most *sl*	greater than *med*	*sl*
	5.5	*sl*	*vl*	(1.8,0.5,0.05)
	low	[3.5,4.5]	*1*	better than *vl*
	[2,3]	between *sl* and *sh*	*med*	(2.8,0.5,0.05)
		[5,7]	[0,2]	lower than *sl*
			between *low* and *sh*	(3.8,0.5,0.05)
	high	[0,0.5]	between *low* and *sl*	between *low* and *sl*
	lower than *sl*	[6-9]	lower than *sl*	*sl*
	between *low* and *med*	at most *med*	*med*	lower than *sh*
	between *med* and *hign*	(5,1,0.04)	lower than *sl*	4
	vl	*med*	between *low* and *sl*	lower than *sl*
	vl	*med*	*sl*	0
S	[4,5]	(8,1,0.2)	at most *sh*	between *sl* and *sh*
	between *vl* and *med*	at most *med*	greater than *med*	*sl*
	5.5	sl	*vl*	(3.8,0.5,0.05)
	low	[3.5,4.5]	1	better than *vl*
	[5,6]	between sl and sh	*med*	(2.8,0.5,0.05)
		[5,7]	[4,9]	lower than *sl*
			between *low* and *sh*	(3.8,0.5,0.05)
	med	[5,7]	between *low* and *sl*	*m*
	between *sl* and *sh*	6	lower than *high*	*high*
	lower than *high*	greater than *sh*	*high*	greater than *sh*
	between *med* and *sh*	(5,1,0.04)	at most *sh*	8
	sh	*med*	7.8	between *low* and *med*
	vl	greater than *sh*	*sh*	20
D	[5,7]	(0.5,1,0.2)	between *low* and *med*	between *vl* and *vh*
	between *med* and *sh*	at most *sh*	greater than *sh*	*high*
	5.5	greater than *sh*	*sh*	[6,7]
	sl	[3.5,4.5]	3	between *med* and *sh*
	[5,6]	between *sl* and *sh*	*med*	[5,8]
		[5,7]	[4,8]	better than *sh*
			between *low* and *sh*	(5.8,0.5,0.03)

Table S11. The NCM representations of original data on FM_4

RF	Subgroup			
	G_1	G_2	G_3	G_4
	(8.7430,1.2420,0.0410)	(0.2500,0.0833,0.0000)	(2.1645,1.3522,0.0474)	(2.1645,1.3522,0.0474)
	(0.4810,1.0620,0.0452)	/ [a]	(0.4810,1.0620,0.0452)	(3.0830,1.0300,0.0330)
	(3.1290,1.7208,0.0734)	(1.9168,1.7208,0.0792)	(5.0580,1.1090,0.0560)	(1.9168,1.7208,0.0792)
	(4.0705,1.3987,0.0650)	(3.0000,1.0000,0.0400)	(1.9168,1.7208,0.0792)	(4.0000,0.0000,0.0000)
	(0.1970,0.4570,0.0220)	(5.0580,1.1090,0.0560)	(2.1645,1.3522,0.0474)	(0.4810,1.0620,0.0452)
	(0.1970,0.4570,0.0220)	(3.0830,1.0300,0.0330)	(3.0830,1.0300,0.0330)	/
O	(3.0000,0.6667,0.0000)	(8.0000,1.0000,0.2000)	(2.7815,2.0210,0.0891)	(5.0820,1.6988,0.0769)
	(2.3960,1.7208,0.0766)	(1.1315,1.3522,0.0559)	(8.9058,1.4855,0.0670)	(3.0830,1.0300,0.0330)
	(5.5000,0.0000,0.0000)	(3.0830,1.0300,0.0330)	(0.1970,0.4570,0.0220)	(1.8000,0.5000,0.0500)
	(1.2460,1.0620,0.0340)	(4.0000,0.1667,0.0000)	(1.0000,0.0000,0.0000)	/
	(2.5000,0.1667,0.0000)	(5.0820,1.6988,0.0769)	(5.0580,1.1090,0.0560)	(2.8000,0.5000,0.0500)
		(6.0000,0.3333,0.0000)	(1.0000,0.3333,0.0000)	(0.4810,1.0620,0.0452)
			(4.1230,2.0210,0.0840)	(3.8000,0.5000,0.0500)
	(8.7430,1.2420,0.0410)	(0.2500,0.0833,0.0000)	(2.1645,1.3522,0.0474)	(2.1645,1.3522,0.0474)
	(0.4810,1.0620,0.0452)	/	(0.4810,1.0620,0.0452)	(3.0830,1.0300,0.0330)
	(3.1290,1.7208,0.0734)	(1.9168,1.7208,0.0792)	(5.0580,1.1090,0.0560)	(1.9168,1.7208,0.0792)
	(5.0580,1.1090,0.0560)	(5.0000,1.0000,0.0400)	(0.4810,1.0620,0.0452)	(4.0000,0.0000,0.0000)
	(0.1970,0.4570,0.0220)	(5.0580,1.1090,0.0560)	(2.1645,1.3522,0.0474)	(0.4810,1.0620,0.0452)
	(0.1970,0.4570,0.0220)	(5.0580,1.1090,0.0560)	(3.0830,1.0300,0.0330)	(0.0000,0.0000,0.0000)
S	(4.5000,0.1667,0.0000)	(8.0000,1.0000,0.2000)	(2.7815,2.0210,0.0891)	(5.0820,1.6988,0.0769)
	(2.3960,1.7208,0.0766)	(1.9168,1.7208,0.0792)	(8.9058,1.4855,0.0670)	(3.0830,1.0300,0.0330)
	(5.5000,0.0000,0.0000)	(3.0830,1.0300,0.0330)	(0.1970,0.4570,0.0220)	(3.8000,0.5000,0.0500)
	(1.2460,1.0620,0.0340)	(4.0000,0.1667,0.0000)	(1.0000,0.0000,0.0000)	/
	(5.5000,0.1667,0.0000)	(5.0820,1.6988,0.0769)	(5.0580,1.1090,0.0560)	(2.8000,0.5000,0.0500)
		(6.0000,0.3333,0.0000)	(6.5000,0.8333,0.0000)	(0.4810,1.0620,0.0452)
			(4.1230,2.0210,0.0840)	(3.8000,0.5000,0.0500)
	(5.0580,1.1090,0.0560)	(6.0000,0.3333,0.0000)	(2.1645,1.3522,0.0474)	(10.0000,0.1000,0.0200)
	(5.0820,1.6988,0.0769)	(6.0000,0.0000,0.0000)	(2.7815,2.0210,0.0891)	(8.7430,1.2420,0.0410)
	(2.7815,2.0210,0.0891)	(9.5060,1.3200,0.0530)	(8.7430,1.2420,0.0410)	(9.5060,1.3200,0.0530)
	(6.0815,1.4092,0.0694)	(5.0000,1.0000,0.0400)	(2.7815,2.0210,0.0891)	(8.0000,0.0000,0.0000)
	(7.1050,1.0270,0.0410)	(5.0580,1.1090,0.0560)	(7.8000,0.0000,0.0000)	(3.1290,1.7208,0.0734)
	(0.1970,0.4570,0.0220)	(9.5060,1.3200,0.0530)	(7.1050,1.0270,0.0410)	/
D	(6.0000,0.3333,0.0000)	(0.5000,1.0000,0.2000)	(3.1290,1.7208,0.0734)	(5.0296,2.4795,0.0998)
	(6.0815,1.4092,0.0694)	(2.7815,2.0210,0.0891)	(9.5060,1.3200,0.0530)	(8.7430,1.2420,0.0410)
	(5.5000,0.0000,0.0000)	(9.5060,1.3200,0.0530)	(7.1050,1.0270,0.0410)	(6.5000,0.1667,0.0000)
	(3.0830,1.0300,0.0330)	(4.0000,0.1667,0.0000)	(3.0000,0.0000,0.0000)	(6.0815,1.4092,0.0694)
	(5.5000,0.1667,0.0000)	(5.0820,1.6988,0.0769)	(5.0580,1.1090,0.0560)	(6.5000,0.5000,0.0000)
		(6.0000,0.3333,0.0000)	(6.0000,0.6667,0.0000)	/
			(4.1230,2.0210,0.0840)	(5.8000,0.5000,0.0300)

[a] The forward slash (/) indicates an invalid data at this location, and therefore it was not converted into an NCM.

Table S12. The data weights and comprehensive weights on FM_4

RF	Subgroup							
	G_1		G_2		G_3		G_4	
	Data weights	Comprehensive	Data weights	Comprehensive	Data weights	Comprehensive	Data weights	Comprehensive
	/ [a]	/	0.1081	0.0170	0.3718	0.0241	0.3196	0.0190
	0.2423	0.0248	/	/	0.2585	0.0203	0.4127	0.0221
	0.1039	0.0202	0.1639	0.0188	0.3870	0.0246	0.1436	0.0131
	0.1844	0.0229	0.4866	0.0296	0.2363	0.0196	0.6247	0.0292
	0.2809	0.0261	0.3270	0.0243	0.3871	0.0246	0.1452	0.0132
	0.2809	0.0261	0.4424	0.0281	0.5226	0.0292	/	/
O	0.5916	0.0365	/	/	0.1577	0.0170	0.1215	0.0124
	0.1040	0.0202	0.2144	0.0205	/	/	0.4494	0.0234
	0.5276	0.0343	0.4527	0.0285	0.1680	0.0173	0.4489	0.0233
	0.3536	0.0285	0.7477	0.0383	0.3663	0.0239	/	/
	0.6748	0.0392	0.1660	0.0189	0.3516	0.0234	0.6245	0.0292
			/	/	0.5580	0.0303	0.1461	0.0132
					0.1494	0.0167	0.5797	0.0277
	/	/	0.0572	0.0150	0.3522	0.0230	0.2309	0.0158
	0.2034	0.0231	/	/	0.2814	0.0207	0.3740	0.0205
	0.0714	0.0187	0.1234	0.0172	0.4252	0.0254	0.0906	0.0112
	0.2541	0.0247	0.4601	0.0282	0.2890	0.0210	0.5710	0.0269
	0.2962	0.0261	0.3571	0.0248	0.3820	0.0240	0.1008	0.0115
	0.2962	0.0261	0.3571	0.0248	0.5154	0.0284	/	/
S	0.6794	0.0387	/	/	0.1464	0.0163	0.0849	0.0110
	0.0684	0.0186	0.1240	0.0172	/	/	0.3976	0.0212
	0.6448	0.0376	0.4432	0.0277	0.2314	0.0191	0.5067	0.0248
	0.2833	0.0257	0.7471	0.0376	0.3697	0.0236	/	/
	0.6430	0.0375	0.1686	0.0187	0.4041	0.0247	0.5363	0.0258
		0.0000	0.7697	0.0384	0.4418	0.0260	0.0882	0.0111
		0.0000		0.0000	0.1484	0.0164	0.5141	0.0251
	0.4683	0.0289	0.7973	0.0357	0.1710	0.0155	0.4084	0.0196
	0.2043	0.0210	0.7964	0.0357	0.1228	0.0141	0.2213	0.0141
	0.0077	0.0151	0.1458	0.0163	0.3212	0.0200	0.1793	0.0128
	0.2976	0.0238	0.4788	0.0262	0.1228	0.0141	0.6884	0.0280
	0.3577	0.0256	0.4096	0.0242	0.5800	0.0277	0.0071	0.0077
	/	0.0000	0.1921	0.0177	0.4954	0.0252	/	/
D	0.7225	0.0365	/	/	0.2278	0.0172	/	/
	0.2976	0.0238	0.0102	0.0122	0.2455	0.0178	0.2277	0.0142
	0.7225	0.0365	0.1911	0.0176	0.4954	0.0252	0.8074	0.0315
	0.1232	0.0186	0.4432	0.0252	0.4291	0.0232	0.1369	0.0115
	0.7554	0.0375	0.1629	0.0168	0.5295	0.0262	0.7504	0.0298
			0.8059	0.0360	0.6923	0.0311	/	/
					0.1863	0.0160	0.6353	0.0264

[a] The forward slash (/) signifies removed data at this position, so its weight was not calculated.

LGDM results for O, S, and D were calculated as (2.6459,0.1562,0.0067), (3.2843,0.1470,0.0065), and (6.0324,0.1448,0.0062) respectively. The RPN value of FM_4 was calculated as (52.4222,4.0819,0.1767) by multiplying O, S, and D.

V. DATA ON FM_5

Table S13. The original data provided by experts on FM_5

RF	Subgroups and original data			
	G_1	G_2	G_3	G_4
O	*sl*	[0,0.5]	between *low* and *sl*	between *med* and *sh*
	between *sl* and *med*	5	lower than *med*	*sl*
	between *low* and *med*	between *low* and *sh*	*med*	lower than *sh*
	between *low* and *med*	(6,1,0.04)	at most *sh*	4
	sh	*med*	5	between *low* and *med*
	vl	between *low* and *sl*	*sl*	at most *sl*
	[3,7]	(5,1,0.2)	between *low* and *med*	between *vl* and *sh*
	between *med* and *sh*	at most *sh*	greater than *vh*	*sl*
	[3,7]	*sl*	*med*	[2,7]
	at most *sl*	[3.5,4.5]	5	between *sl* and *sh*
	[2,6]	between *sl* and *sh*	*med*	[5,8]
		at most *sl*	[4,8]	better than *sh*
S	[2,8]	between *sh* and *high*	*sh*	*sh*
	sh	*sh*	between *sl* and *sh*	*sl*
	between *sl* and *sh*	1.5	*sh*	between *low* and *sh*
	sh	*med*	*high*	[5,8]
	(6,1,0.1)	[5,8]	*high*	*sh*
	sh	at least *sh*	between *sh* and *sh*	(7.5,0.5,0.1)
	sh	at most *med*	(5,1,0.2)	*low*
	[4,8]	5	10	*sh*
	5	*sl*		*high*
	sh	[4,6]	between *sl* and *sh*	*sh*
	between *sh* and *sh*	*sh*	*sl*	*sh*
	7		*sh*	[7,8]
D	*sl*	[0,0.5]	between *low* and *sl*	between *sl* and *med*
	between *sl* and *med*	4	lower than *med*	*sl*
	between *low* and *med*	between *low* and *med*	*med*	lower than *med*
	between *low* and *med*	(6,1,0.04)	at most *med*	4
	med	*med*	5	between *low* and *med*
	vl	between *low* and *sl*	*med*	6
	[3,7]	(5.5,1,0.2)		between *vl* and *med*
	between *low* and *med*	at most *med*	greater than *vh*	*sl*
	[3,7]	*med*	*sl*	[6,7]
	med	[3.5,4.5]	3	between *sl* and *med*
	[2,6]	between *sl* and *med*	*med*	[5,7]
		(4.9,1,0.04)	[4,8]	better than *med*

Table S14. The NCM representations of original data on FM_5

RF	Subgroup			
	G_1	G_2	G_3	G_4
O	(3.0830,1.0300,0.0330)	(0.2500,0.0833,0.0000)	(2.1645,1.3522,0.0474)	(6.0815,1.4092,0.0694)
	(4.0705,1.3987,0.0650)	(5.0000,0.0000,0.0000)	(1.1315,1.3522,0.0559)	(3.0830,1.0300,0.0330)
	(3.1290,1.7208,0.0734)	(4.1230,2.0210,0.0840)	(5.0580,1.1090,0.0560)	(1.9168,1.7208,0.0792)
	(3.1290,1.7208,0.0734)	(6.0000,1.0000,0.0400)	(2.7815,2.0210,0.0891)	(4.0000,0.0000,0.0000)
	(7.1050,1.0270,0.0410)	(5.0580,1.1090,0.0560)	(5.0000,0.0000,0.0000)	(3.1290,1.7208,0.0734)
	(0.1970,0.4570,0.0220)	(2.1645,1.3522,0.0474)	(3.0830,1.0300,0.0330)	(1.1315,1.3522,0.0559)
	(5.0000,0.6667,0.0000)	(5.0000,1.0000,0.2000)	(3.1290,1.7208,0.0734)	(3.3378,2.0210,0.0869)
	(6.0815,1.4092,0.0694)	(2.7815,2.0210,0.0891)	(10.0000,0.1000,0.0200)	(3.0830,1.0300,0.0330)
	(5.0000,0.6667,0.0000)	(3.0830,1.0300,0.0330)	(5.0580,1.1090,0.0560)	(4.5000,0.8333,0.0000)
	(1.1315,1.3522,0.0559)	(4.0000,0.1667,0.0000)	(5.0000,0.0000,0.0000)	(5.0820,1.6988,0.0769)
	(4.0000,0.6667,0.0000)	(5.0820,1.6988,0.0769)	(5.0580,1.1090,0.0560)	(6.5000,0.5000,0.0000)
		(1.1315,1.3522,0.0559)	(6.0000,0.6667,0.0000)	/ [a]
S	(5.0000,1.0000,0.0000)	(7.9240,1.4075,0.0580)	(7.1050,1.0270,0.0410)	(7.1050,1.0270,0.0410)
	(7.1050,1.0270,0.0410)	(7.1050,1.0270,0.0410)	(5.0820,1.6988,0.0769)	(3.0830,1.0300,0.0330)
	(5.0820,1.6988,0.0769)	(1.5000,0.0000,0.0000)	(7.1050,1.0270,0.0410)	(4.1230,2.0210,0.0840)
	(7.1050,1.0270,0.0410)	(5.0580,1.1090,0.0560)	(8.7430,1.2420,0.0410)	(6.5000,0.5000,0.0000)
	(6.0000,1.0000,0.1000)	(6.5000,0.5000,0.0000)	(8.7430,1.2420,0.0410)	(7.1050,1.0270,0.0410)
	(7.1050,1.0270,0.0410)	(8.9058,1.4855,0.0670)	(7.1050,1.0270,0.0410)	(7.5000,0.5000,0.1000)
	(7.1050,1.0270,0.0410)	(1.9168,1.7208,0.0792)	(5.0000,1.0000,0.2000)	(1.2460,1.0620,0.0340)
	(6.0000,0.6667,0.0000)	(5.0000,0.0000,0.0000)	(10.0000,0.0000,0.0000)	(7.1050,1.0270,0.0410)
	(5.0000,0.0000,0.0000)	(3.0830,1.0300,0.0330)	/	(8.7430,1.2420,0.0410)
	(7.1050,1.0270,0.0410)	(5.0000,0.3333,0.0000)	(5.0820,1.6988,0.0769)	(7.1050,1.0270,0.0410)
	(7.1050,1.0270,0.0410)	(7.1050,1.0270,0.0410)	(3.0830,1.0300,0.0330)	(7.1050,1.0270,0.0410)
	(7.0000,0.0000,0.0000)		(7.1050,1.0270,0.0410)	(7.5000,0.1667,0.0000)
D	(3.0830,1.0300,0.0330)	(0.2500,0.0833,0.0000)	(2.1645,1.3522,0.0474)	(4.0705,1.3987,0.0650)
	(4.0705,1.3987,0.0650)	(4.0000,0.0000,0.0000)	(1.1315,1.3522,0.0559)	(3.0830,1.0300,0.0330)
	(3.1290,1.7208,0.0734)	(3.1290,1.7208,0.0734)	(5.0580,1.1090,0.0560)	(1.1315,1.3522,0.0559)
	(3.1290,1.7208,0.0734)	(6.0000,1.0000,0.0400)	(1.9168,1.7208,0.0792)	(4.0000,0.0000,0.0000)
	(5.0580,1.1090,0.0560)	(5.0580,1.1090,0.0560)	(5.0000,0.0000,0.0000)	(3.1290,1.7208,0.0734)
	(0.1970,0.4570,0.0220)	(2.1645,1.3522,0.0474)	(5.0580,1.1090,0.0560)	(6.0000,0.0000,0.0000)
	(5.0000,0.6667,0.0000)	(5.5000,1.0000,0.2000)	/	(2.3960,1.7208,0.0766)
	(3.1290,1.7208,0.0734)	(1.9168,1.7208,0.0792)	(10.0000,0.1000,0.0200)	(3.0830,1.0300,0.0330)
	(5.0000,0.6667,0.0000)	(5.0580,1.1090,0.0560)	(3.0830,1.0300,0.0330)	(6.5000,0.1667,0.0000)
	(5.0580,1.1090,0.0560)	(4.0000,0.1667,0.0000)	(3.0000,0.0000,0.0000)	(4.0705,1.3987,0.0650)
	(4.0000,0.6667,0.0000)	(4.0705,1.3987,0.0650)	(5.0580,1.1090,0.0560)	(6.0000,0.3333,0.0000)
		(4.9000,1.0000,0.0400)	(6.0000,0.6667,0.0000)	/

[a] The forward slash (/) indicates an invalid data at this location, and therefore it was not converted into an NCM.

Table S15. The data weights and comprehensive weights on FM_5

RF	Subgroup							
	G_1		G_2		G_3		G_4	
	Data weights	Comprehensive	Data weights	Comprehensive	Data weights	Comprehensive	Data weights	Comprehensive
	0.5127	0.0324	/	/	0.2146	0.0181	0.2468	0.0159
	0.3984	0.0288	0.6546	0.0338	0.0211	0.0119	0.4626	0.0228
	0.2443	0.0238	0.1831	0.0187	0.4350	0.0251	0.1762	0.0136
	0.2443	0.0238	0.4267	0.0265	0.0927	0.0142	0.6124	0.0276
	0.2864	0.0252	0.4363	0.0268	0.6432	0.0318	0.2985	0.0176
O	/ [a]	/	0.3207	0.0231	0.5017	0.0273	0.0483	0.0095
	0.6680	0.0374	/	/	0.2022	0.0177	0.1873	0.0140
	0.2898	0.0253	0.1520	0.0177	/	/	0.5495	0.0256
	0.6680	0.0374	0.5571	0.0306	0.4360	0.0252	0.6703	0.0295
	0.0091	0.0163	0.4741	0.0280	0.6361	0.0316	0.2760	0.0168
	0.7105	0.0388	0.2679	0.0214	0.4219	0.0247	0.4545	0.0226
			0.0531	0.0145	0.4926	0.0270	/	/
	0.0709	0.0255	/	/	0.1573	0.0227	0.2952	0.0243
	0.3209	0.0367	0.1481	0.0245	/	/	/	/
	/	0.0000	/	/	0.6254	0.0436		0.0000
	0.3209	0.0367	0.1197	0.0232	0.1430	0.0220	0.3902	0.0286
	/	/	0.7270	0.0503	0.3514	0.0313	0.4206	0.0300
S	0.3209	0.0367	/	/	0.5015	0.0380	/	/
	0.3209	0.0367	/	/	/	/	/	/
	0.5914	0.0487	0.5878	0.0441	/	/	0.3195	0.0254
	0.0650	0.0252	/	/	/	/	0.0120	0.0117
	0.3209	0.0367	0.4926	0.0399	/	/	0.3944	0.0288
	0.3209	0.0367	0.1481	0.0245	/	/	0.4823	0.0327
	0.6714	0.0523			0.4487	0.0357	0.8034	0.0470
	0.3600	0.0284	/	/	0.2436	0.0196	0.2370	0.0161
	0.4143	0.0302	0.6664	0.0353	0.0541	0.0134	0.3603	0.0202
	0.1707	0.0222	0.1829	0.0193	0.3631	0.0236	0.0271	0.0092
	0.1707	0.0222	0.3325	0.0242	0.0775	0.0141	0.6344	0.0293
	0.3861	0.0293	0.4185	0.0271	0.6392	0.0327	0.1385	0.0129
D	/	/	0.1519	0.0183	0.3865	0.0244	0.5882	0.0277
	0.4853	0.0326	/	/	/	/	0.1085	0.0119
	0.1707	0.0222	0.0357	0.0144	/	/	0.4136	0.0220
	0.4853	0.0326	0.4192	0.0271	0.4455	0.0263	0.4726	0.0239
	0.3861	0.0293	0.6933	0.0362	0.6256	0.0323	0.2494	0.0165
	0.6764	0.0389	0.3506	0.0248	0.3610	0.0235	0.5953	0.0280
			0.5239	0.0306	0.4811	0.0275	/	/

[a] The forward slash (/) signifies removed data at this position, so its weight was not calculated.

LGDM results for O, S, and D were calculated as (4.1659,0.1737,0.0070), (6.7920,0.1592,0.0059), and (4.1813,0.1599,0.0068) respectively. The RPN value of FM_5 was calculated as (118.3090,7.2450,0.2954) by multiplying O, S, and D.
